# Characterization of the ovine ribosomal protein SA gene and its pseudogenes

**DOI:** 10.1186/1471-2164-11-179

**Published:** 2010-03-16

**Authors:** Alice Van den Broeke, Mario Van Poucke, Ane Marcos-Carcavilla, Karine Hugot, Hélène Hayes, Maud Bertaud, Alex Van Zeveren, Luc J Peelman

**Affiliations:** 1 Department of Nutrition, Genetics and Ethology, Faculty of Veterinary Medicine, Ghent University, Heidestraat 19, B-9820 Merelbeke, Belgium; 2 Departamento de Mejora Genética Animal, INIA, Ctra La Coruña Km 7.5, Madrid 28040, Spain; 3 INRA, UMR 1313 Génétique Animale et Biologie Intégrative, F78350 Jouy-en-Josas, France

## Abstract

**Background:**

The ribosomal protein SA (RPSA), previously named 37-kDa laminin receptor precursor/67-kDa laminin receptor (LRP/LR) is a multifunctional protein that plays a role in a number of pathological processes, such as cancer and prion diseases. In all investigated species, *RPSA *is a member of a multicopy gene family consisting of one full length functional gene and several pseudogenes. Therefore, for studies on RPSA related pathways/pathologies, it is important to characterize the whole family and to address the possible function of the other *RPSA *family members. The present work aims at deciphering the *RPSA *family in sheep.

**Results:**

In addition to the full length functional ovine *RPSA *gene, 11 other members of this multicopy gene family, all processed pseudogenes, were identified. Comparison between the *RPSA *transcript and these pseudogenes shows a large variety in sequence identities ranging from 99% to 74%. Only one of the 11 pseudogenes, i.e. *RPSAP7*, shares the same open reading frame (ORF) of 295 amino acids with the *RPSA *gene, differing in only one amino acid. All members of the *RPSA *family were annotated by comparative mapping and fluorescence *in situ *hybridization (FISH) localization. Transcription was investigated in the cerebrum, cerebellum, spleen, muscle, lymph node, duodenum and blood, and transcripts were detected for 6 of the 11 pseudogenes in some of these tissues.

**Conclusions:**

In the present work we have characterized the ovine *RPSA *family. Our results have revealed the existence of 11 ovine *RPSA *pseudogenes and provide new data on their structure and sequence. Such information will facilitate molecular studies of the functional *RPSA *gene taking into account the existence of these pseudogenes in the design of experiments. It remains to be investigated if the transcribed members are functional as regulatory non-coding RNA or as functional proteins.

## Background

The ribosomal protein SA (RPSA), previously named 37-kDa laminin receptor precursor/67-kDa laminin receptor (LRP/LR) is a multifunctional protein. In the nucleus it binds to DNA via the histones H2A, H2B and H4 [1], in the cytoplasm it is associated with the 40S ribosomal subunit [2], and at the cell surface it acts as a receptor for a number of components i.e. laminin, elastin, the green tea catechin epigallocatechin-3-gallate (EGCG), carbohydrates, the prion protein, different viruses like Dengue virus, Sindbis virus, Venezuelean Equine Encephalitis virus and Adeno-associated-viruses and various bacteria like Streptococcus pneumoniae, Neisseria meningitidis and Haemophilus influenza [2,3].

The receptor is involved in many pathological processes. It is upregulated in cancer and its expression is positively correlated with metastasis and the aggressiveness of tumour cells in breast, ovary, lung, prostate and cervical carcinomas [2]. In the context of prion disease, RPSA is needed for the internalization and propagation of prion proteins [2]. Several therapeutic approaches based on down-regulation (e.g. via RNA interference) and/or blocking (e.g. with specific antibodies or trans-dominant negative mutants) of the receptor result in reduced adhesion, migration and invasion of tumour cells [4-7], and reduced accumulation of the pathogenic isoform of the prion protein in many organs involved in the pathogenesis of transmissible spongiform encephalopathies [8-12], leading to a significant prolongation of the pre-clinical phase or survival time after the occurrence of the first symptoms [10-12].

In addition, it has been shown that binding of green tea catechin EGCG to RPSA causes anti-thrombotic, anti-allergic and anti-obesity effects and mediates cancer prevention by inhibiting cell growth [13-16], thus RPSA is a target in new therapies against this large group of diseases.

However, in order to unravel the multiple pathways in which RPSA is involved and to develop RPSA-based diagnostic/therapeutic tools, it is necessary first to characterize in full detail the complex genetic background of RPSA. Indeed, previous studies have shown that in most investigated species thus far, *RPSA *is a member of a multicopy gene family consisting of one full length functional gene and several pseudogenes (e.g. at least 63 in man; Table 1). Moreover, the presence of pseudogenes in a genome can interfere with molecular studies of the corresponding functional gene (i.e. sequencing, mapping, polymorphism detection, genotyping, association analysis, mRNA expression studies, ...) and transcribed pseudogenes can produce endogenous small interfering RNAs that regulate the expression of the functional gene or other genes [17].

**Table 1:** Number of *RPSA *pseudogenes in different species identified so far

Species	Processed pseudogenes/transcribed	Duplicated pseudogenes	Reference
*Homo sapiens*	63^(a)^/1^(b)^	/	Balasubramanian et al. (2009) [45]^(a)^ Asano et al. (2004)[46]^(b)^

*Bos taurus*	60^(c)^/1^(b)^	/	Germerodt et al. (2004) [32]^(b)^

*Sus scrofa*	2^(b)^	1^(b)^	Knorr et al. (2007) [47]^(b)^

*Mus musculus*	45^(a)^/2^(b)^	/	Balasubramanian et al. (2009) [45]^(a)^ Fernandez et al. (1991) [48]^(b)^

*Gallus gallus*	/	/	Bignon et al. (1991) [49]^(b)^

*Ovis aries*	/	1^(b)^?	Marcos-Carcavilla et al. (2008)[18]^(b)^

*Pan troglodytes*	52^(a)^		Balasubramanian et al. (2009) [45]^(a)^

*Rattus norvegicus*	45^(a)^		Balasubramanian et al. (2009)[45]^(a)^

^(a) ^*In silico *genome-wide screening studies in species with fully sequenced genomes. ^(b) ^*In vitro *studies screening genomic or cDNA library, ^(c) ^*In silico *genome-wide screening study carried out in this paper.

Previously, Marcos-Carcavilla et al. [18] have postulated the existence of an ovine *RPSA *pseudogene. The present work aims at providing a genetic basis for future studies on RPSA related pathways/pathologies in sheep by identifying and characterizing the complex *RPSA *gene family.

## Results and Discussion

### BAC screening and STS content mapping

**Figure 1:**
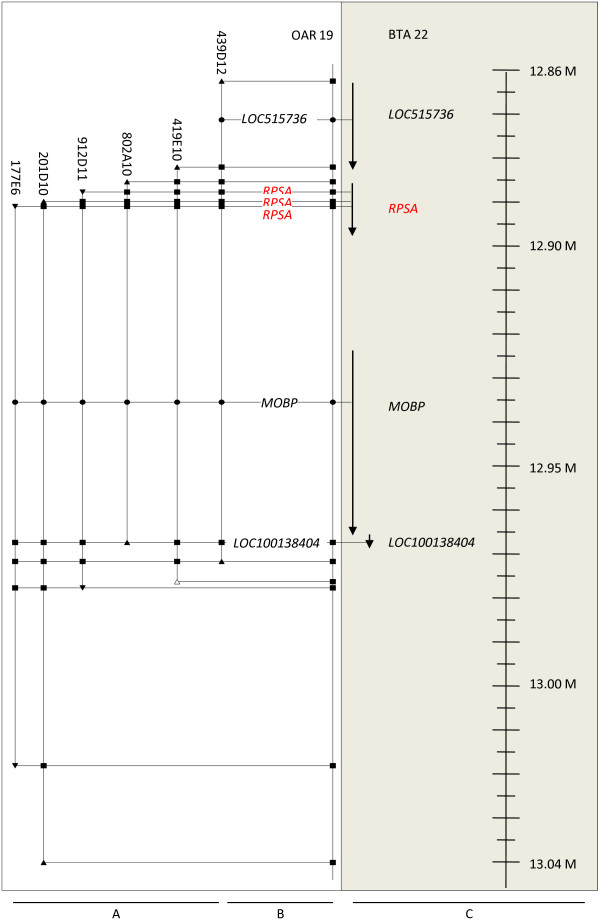
**Comparative mapping of the region of *RPSA *in sheep and cattle**. The ovine BAC mini-contig is drawn in part A. Triangles represent BAC end sequences; pointing towards the 3'-end of the BAC clone. Black triangles represent BES from which primers were designed to construct the mini-contig. White triangles are BES from which it was impossible to design STS-primers. Black squares show overlaps between BES and other BAC clones. Black circles represent genes annotated by PCR. Annotated sequences are shown in a plane map in part B. The position and orientation of the genes present in the syntenic region of *Bos taurus *are represented with arrows (C).

**Figure 2:**
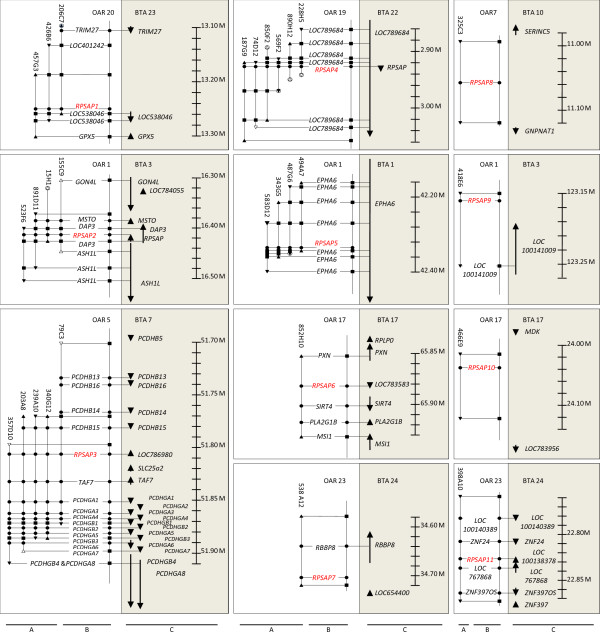
**Comparative mapping of the region of 11 *RPSA *pseudogenes in sheep and cattle**. The ovine BAC mini-contigs are drawn in part A. Triangles represent BAC end sequences; pointing towards the 3'-end of the BAC clone. Black triangles represent BES from which primers were designed to construct the mini-contigs. White triangles are BES from which it was impossible to design STS-primers. Encircled triangles represent BES that are not annotated. Black squares show overlaps between BES and other BAC clones. Black circles represent genes annotated by PCR. Annotated sequences are shown in a plane map in part B. The position and orientation of the genes present in the syntenic region of *Bos taurus *are represented with arrows (C).

Eight different primer pairs were designed in conserved ovine *RPSA *regions identified by aligning previously described mRNA and expressed sequence tag (EST) sequences, representing each exon at least once. Using these primers, 34 bacterial artificial chromosome (BAC) clones, containing members of the *RPSA *family, were isolated by PCR screening of the INRA sheep BAC library [19], with an annealing temperature (Ta) that was at least 8°C lower than the melting temperature (Tm) of the primers to allow primer mismatches (Additional file 1). By sequence tagged site (STS) content mapping, performed with 54 unique STS primer pairs that were designed from the 68 BAC end sequences (BES) [GenBank:GS375851-GS375918], 6 mini-contigs could be constructed and another 6 single BAC clones could be identified, each containing a different family member of the ovine *RPSA *family (Figure 1 and 2; Additional file 2).

### Characterization of the 12 *RPSA *gene family members

Each member of the *RPSA *gene family was sequenced by direct sequencing on BAC DNA starting with the PCR primers as sequencing primers and finishing by primer walking. The sequences were assembled with the CAP3 program [20] and annotated with BLAST [21].

The full length functional gene, that was first described by Marcos-Carcavilla et al. [18], was present in one of the contigs composed of 6 BAC clones [Genbank:GQ202529]. We have sequenced for the first time, the complete intron 3, comprising 8846 bp, which like the other introns, has consensus acceptor and donor splice sites. The full length ovine *RPSA *gene consist thus of 13287 bp.

**Figure 3:**
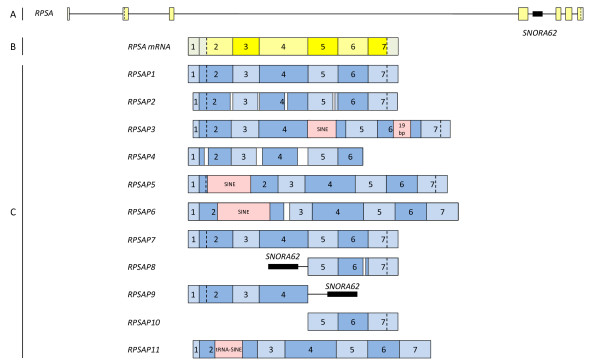
**Schematic overview of the *RPSA *(pseudo)genes**. The genomic structure of the ovine *RPSA *gene is drawn in part A, the mRNA of the *RPSA *gene is drawn in part B. Part C represents the genomic structure of the different *RPSA *pseudogenes. The squares represent exons and the lines stand for introns. The coding sequence (CDS) is drawn in yellow; the untranslated sequences in green. The blue squares are parts of the pseudogene sequence that are analogous with the exons of the *RPSA *mRNA. The pink squares symbolize interspersed sequences and the white gaps deletions. *SNORA62 *is represented as a black square. Start codons and stop codons, analogous with the ones of *RPSA*, are represented by a dotted line.

Besides the full length functional gene, 11 other *RPSA *gene family members were sequenced [GenBank:GQ202530-GQ202540]. A schematic representation of all the family members, based on sequence alignments with the full length functional gene (Additional file 3), is included in Figure 3. They all are considered as processed pseudogenes and in accordance with *RPSA *pseudogenes described in other species, they have been assigned the names *RPSAP1*-*RPSAP11*. Pseudogenes arise in 2 different manners: either by retrotransposition of the mRNA of the ancestral gene into the genome or by duplication of genomic DNA [22]. The first class is known as processed pseudogenes, the second one as non processed pseudogenes. The majority of the pseudogenes are processed and originate from housekeeping genes, with ribosomal protein genes as largest subgroup [22,23]. As processed pseudogenes are inserted without internal promoter, they are released from selection pressure and accumulate mutations during evolution leading to frameshift mutations and/or premature stopcodons which prevents them of encoding a functional protein [24]. In some cases nevertheless, they have obtained a (regulatory) function [17].

To investigate this possibility, all the *RPSA *pseudogenes were further characterized *in silico *and their main characteristics are listed in detail in Table 2 and 3. Comparison with the full length *RPSA *gene transcript shows that the pseudogenes vary greatly both in structure and sequence identity. These differences range from structurally identical pseudogenes sharing 99% sequence identity (*RPSAP7*) to pseudogenes lacking half of the gene (*RPSAP8*, *RPSAP9 *and *RPSAP10*) or containing many deletions throughout the whole gene sharing a sequence identity of only 74% (*RPSAP2*).

**Table 2:** Characteristics of RPSA (pseudo)genes-general characteristics

Gene	Acc. No. GenBank	% nucleotide identity *RPSA*	Start codon/Stop codon	PolyA-signal	Frameshift mutation	Premature stop codon	Longest ORF = *RPSA *(position) identities/positives
*RPSAP1*	GQ202530	93%	ATG/TAA	Yes	No	Yes	83 aa (1-83) 95%/95%

*RPSAP2*	GQ202531	74%	ATG/TAA	Yes	Yes	Yes	70 aa (34-103) 56%/63%

*RPSAP3*	GQ202532	80%	ATG/TAA	No	Yes	Yes	84 aa (1-84) 82%/87%

*RPSAP4*	GQ202533	83%	No/No	No	Yes	N/A	90 aa (10-99) 48%/53%

*RPSAP5*	GQ202534	90%	ATG/TAA	Yes	Yes	Yes	129 aa (10-138) 91%/92%

*RPSAP6*	GQ202535	80%	No/No	No	Yes	N/A	106 aa (160-265) 65%/70%

*RPSAP7*	GQ202536	99%	ATG/TAA	Yes	No	No	295 aa (1-295) 99%/99%

*RPSAP8*	GQ202537	86%	No/TAA	Yes	Yes	N/A	82 aa (177-258) 80%/80%

*RPSAP9*	GQ202538	98%	ATG/No	No	No	No	171 aa (1-171) 100%/100%

*RPSAP10*	GQ202539	94%	No/TAA	Yes	Yes	N/A	57 aa (174-230) 95%/100%

*RPSAP11*	GQ202540	76%	No/No	Yes	Yes	N/A	107 aa (185-291) 46%/49%

**Table 3:** Characteristics of RPSA (pseudo)genes-repeats and transcription

Gene	Interspersed repeats in gene	Flanking repeats/family/class	Transcription
*RPSAP1*	/	/	/

*RPSAP2*	/	3': L2c/L2/LINE	Cerebrum, cerebellum, spleen, muscle, lymph node and duodenum

*RPSAP3*	/	5': ERV3-16A3_I-int/ERVL/LTR 3': CHR-2A/tRNA-Glu/SINE	Cerebrum

*RPSAP4*	ART2A/RTE-BovB/SINE	5': L1M3/L1/LINE 3': L1M3/L1/LINE and (CA)n/Simple_repeat and L1M3/L1/LINE	Cerebrum, cerebellum and spleen

*RPSAP5*	/	/	Cerebrum

*RPSAP6*	ART2A/RTE-BovB/SINE	3': CHRL/tRNA-Glu/SINEand tRNA-Glu-GAA/tRNA	/

*RPSAP7*	Bov-tA1/BovA/SINE	5': LTR16A2/ERVL/LTR 3': L1MEc/L1/LINE and L1M3/L1/LINE	/

*RPSAP8*	/	/	Cerebrum and cerebellum

*RPSAP9*	/	5': MIR/SINE and MIR/SINE and L1M2/L1/LINE 3': L1M2/L1/LINE and (CATA)n/Simple_repeat	/

*RPSAP10*	/	/	/

*RPSAP11*	/	5': Bov-tA2/BovA/SINE 3': L1M5/L1/LINE	Cerebrum, cerebellum, spleen, muscle, lymph node and duodenum

Analysis of the primer binding sites in the pseudogenes showed that in our experimental design the screening primers could anneal to targets down to 83% sequence identity, even in the case of *RPSAP2*.

All BAC clones and thus all *RPSA *family members were isolated with at least 2 primer pairs and there was no concordance between the number of BACs in a mini-contig and the level of sequence identity with *RPSA*. We conclude that it is most likely that we have isolated all the ovine *RPSA *pseudogenes sharing a high level of sequence identity and that therefore can interfere with the functional *RPSA *gene in genetic studies.

To obtain a first indication of possible functionality, *in silico *ORF and promoter prediction analysis were carried out.

The pseudogene *RPSAP7 *is the only member sharing almost an identical ORF with the full length *RPSA *gene. The only one amino acid difference (amino acid 31: D → G) is located in the intracellular part of the receptor that does not belong to any binding site. All the other pseudogenes either lack the start codon or contain a premature stop codon due to nonsense or frameshift mutations. The size of the potential ORF of the other pseudogenes varies and the largest reaches 171 amino acids sharing 100% identity with *RPSA *(Table 2). Most ORFs lie in the intracellular region of RPSA (amino acid 1-101). In case of *RPSAP6*, *RPSAP8*, *RPSAP10 *and *RPSAP11*, the ORF contains a part of the binding sites of RPSA with PrP (direct binding aa 161-180; indirect binding aa 180-285 [25]), but most of them have a low level of amino acid identity.

*In silico *promoter analysis predicted a possible promoter for *RPSAP1*, *RPSAP2*, *RPSAP4*, *RPSAP8*, *RPSAP9 *and *RPSAP10 *(Additional file 4). A consensus polyadenylation signal is present in 7 of the 11 pseudogenes (including *RPSAP7*).

Repeated sequences were identified with Repeatmasker [26] and showed that 4 pseudogenes are disrupted by interspersed repeats belonging to the class/family SINE/RTE-BovB, SINE/BovA, tRNA and SINE/tRNA-Glu, and that 7 pseudogenes were flanked by repeats belonging to the SINE, LINE, tRNA, LTR and simple repeat classes. According to Zhang et al. [27], processed pseudogenes are mostly found in genomic regions with a relatively low GC content, as do LINE repeats. Thus, it is not surprising that such repeats are present in the regions flanking many of the *RPSA *pseudogenes.

A remarkable observation is that part of the *RPSA *intron 4, containing the small nucleolar RNA (snoRNA) *SNORA62*, is present in the *RPSAP8 *and *RPSAP9 *pseudogenes. Therefore, these pseudogenes can be considered as semi-processed pseudogenes, which are very rarely reported and defined by Zhang et al. as "pseudogenes that contain remnant introns, which suggests that they were derived from semi-processed RNA transcripts" [28].

**Figure 4:**
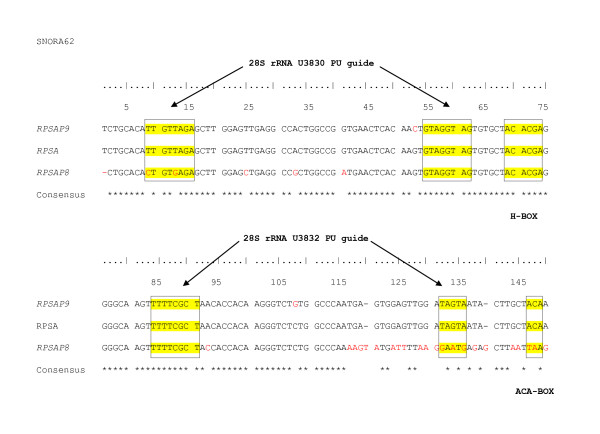
**Alignment of the snoRNAs in *RPSA*, *RPSAP8 *and *RPSAP9***. The ACA-box, H-box and 28S rRNA U3830 and U3832 PU guide are highlighted in yellow.

SnoRNAs are encoded in introns of ribosomal protein genes and other housekeeping genes [29,30], and are responsible for both sequence-specific methylation and pseudouridilation of RNA [31]. *SNORA62 *isan H/ACA box snoRNA that guides the isomerization of uridine into pseudouridine [30] by binding with 2 uridines of 28S rRNA (U3830 and U3832). Sequence comparison shows that these important regions display mutations in *RPSAP8 *but are conserved in *RPSAP9 *(Figure 4). As a result, the paralog of *SNORA62 *is probably not functional in *RPSAP8*. In *RPSAP9 *on the other hand, the paralog of *SNORA62 *could, in addition to *SNORA62*, exhibit the function of pseudouridilation in case of transcription [29]. Marcos-Carcavilla et al. [18] had already postulated the existence of a non-processed pseudogene that differed from the active *RPSA *gene by the absence of a G at position 29 of intron 4. Thus, we hypothesize that this previously mentioned non-processed pseudogene is in fact the semi-processed pseudogene *RPSAP9*, because it lacks the G at position 29 of intron 4 and it can co-amplify with the active *RPSA *gene because of its high sequence identity (98%).

### Annotation of the mini-contigs by comparative mapping and FISH localization

The genomic regions containing the 12 members of the *RPSA *gene family were further investigated by sequence comparison of both BES and internal BAC sequences using NCBI BLAST [21] (Figure 1 and 2). Sixty-two of the 68 BES were annotated while the remaining 6 contained either too many repeat sequences or no specific orthologous sequence to allow annotation. The different characteristics (length, repeat sequences and genes) are listed in the Additional file 5. Based on sequence annotation results, 40 ovine genes, of which 37 have not been described in sheep yet, could be mapped on the mini-contigs by comparative mapping with the bovine genome (Figure 1 and 2). The primers used to perform the PCR for annotating the genes, together with another 18 optimized primer pairs, amplifying genes not present in the mini-contig but flanking the genomic region of the different *RPSA *family members, are listed in Additional file 6.

The 11 pseudogenes were localized by FISH on different sheep chromosomes (see Table 4 and pictures of the FISH experiments in Additional file 7). All the localizations confirmed the positions predicted from the genes present in the mini-contigs by using the online tool Virtual Sheep Genome Assembly v2.0.

As expected, most *RPSA *pseudogenes are located in intergenic regions except 3 found in the intron of other genes (*RPSAP2 *in *DAP3*; *RPSAP4 *in *LOC789684 *and *RPSAP5 *in *EPHA6*; Table 4), which confirms the fact that most processed pseudogenes persist in regions where they do not cause deleterious effects [22].

**Table 4:** Location of *RPSA *(pseudo)genes

Gene	Chromosomal location	Ortholog *Bos taurus*
*RPSA*	OAR19q13 intergenic between *LOC515736 *and *MOBP*	ortholog BTA22: GeneID: 281898

*RPSAP1*	OAR20q22 intergenic between *LOC401242 *and *LOC538046*	no

*RPSAP2*	OAR1p13 in intron 2 *DAP3*	ortholog BTA3: not annotated yet

*RPSAP3*	OAR 5q22.3 intergenic between *PCDHB15 *and *TAF7*	ortholog BTA7: not annotated yet

*RPSAP4*	OAR19q12 in intron 1 *LOC789684*	ortholog BTA22: not annotated yet

*RPSAP5*	OAR1q21-q22 in intron 2 *EPHA6*	no

*RPSAP6*	OAR17q26prox intergenic between *PXN *and *SIRT4*	ortholog BTA17: not annotated yet

*RPSAP7*	OAR23q23prox intergenic between *RBBP8 *and *LOC100138286*	no

*RPSAP8*	OAR7q12-q13 intergenic between *SERINC5 *and *GNPNAT1*	no

*RPSAP9*	OAR1p37 intergenic between *LOC100141009 *and *LOC522241*	no

*RPSAP10*	OAR17q21prox intergenic between *MDK *and *LOC783956*	no

*RPSAP11*	OAR23q21 intergenic between *ZNF24 *and *LOC767868*	ortholog BTA24: GeneID: 100138378

The genomic region around the ovine *RPSA *family members show conserved synteny (same genes, same orientation and same order) with the bovine genome. *LOC784055*, probably a processed pseudogene of *GOLPH3L *located in intron 2 of *GON4L *on *Bos taurus *chromosome (BTA) 3 and expected in the ovine mini-contig containing *RPSAP2*, was the only bovine ortholog not present in sheep and therefore is most probably a bovine specific pseudogene.

The flanking sequences (500 bp upstream and 500 bp downstream) of each *RPSA *pseudogene were blasted against the bovine and human genome. Out of the 11 identified orthologous bovine sequences, 5 were interrupted by a bovine *RPSA *pseudogene; in the 6 other cases, the upstream sequence continued into the downstream sequence without an interruption of a pseudogene. The latter was also the case with the 11 orthologous human sequences. Thus we found 5 orthologous bovine *RPSA *pseudogenes but no human orthologs (Table 5).

**Table 5:** Bovine orthologs

Ovine ortholog	Chromosomal location	GenBank Acc. No.	Range	Nucleic acid identity with bovine *RPSA*	Nucleic acid identity with ovine ortholog	Features in sequence
*RPSA*	BTA22	NC_007320.3	12885045-12898467	100%	CDS 96% Gene 87%	*RPSA*: 12886519-12898467

*RPSAP2*	BTA3	NC_007301.3	16411109-16410143	75%	91%	*DAP3 *intron 2: 16430465-16408264

*RPSAP3*	BTA7	NC_007305.3	51802201-51801057	81%	95%	*LOC786980*: 51801077-51802181

*RPSAP4*	BTA22	NC_007320.3	2925069-2925867	78%	91%	*LOC789684*: 2840530-3050478

*RPSAP6*	BTA17	NC_007315.3	65883767-65884909	81%	92%	*LOC783583*: 65881027-65884941

*RPSAP11*	BTA24	NC_007325.3	22831525-22830341	77%	91%	*LOC100138378*: 22830405-22834810

A BLAST analysis of the bovine genome (reference assembly, based on Btau_4.0) with both ovine and bovine *RPSA *and *RPSA *pseudogene sequences identified 60 potential *RPSA *family members (Additional file 8). These included the only bovine pseudogene described so far, designated as *RPSAP1 *and located on BTA4 [32]. No ortholog of this pseudogene was found in sheep. Twenty-five sequences were annotated as 'similar to Ribosomal protein SA pseudogene' but only one corresponded to an ovine ortholog i.e. *RPSAP11*. To date, the 35 remaining sequences have not been annotated, but we have identified an ovine ortholog in 4 cases (Table 4 and 5; Additional file 8). Apart from *RPSAP3*, the ORF of the ovine and bovine orthologs differ substantially, suggesting that there is no selective pressure to conserve the ORF of these pseudogenes.

No bovine ortholog was found for the 6 sheep *RPSA *pseudogenes sharing 86 to 99% nucleotide identity with *RPSA *whereas the 5, for which a bovine ortholog was identified, only displayed 74-83% sequence identity with *RPSA*. As the amount of mutations accumulated by the pseudogenes during evolution can be used to infer their age [27], it's not surprising that the first group, consisting of recently arisen pseudogenes which have not yet accumulated many mutations, is lineage specific and that the pseudogenes of the latter group, comprising the oldest pseudogenes, all have a bovine ortholog. In addition, none of the 11 ovine pseudogenes were orthologous with any of the 63 annotated human *RPSA *pseudogenes. As a result, we can conclude that all 11 ovine *RPSA *pseudogenes detected originated after the divergence between primates and ungulates and 6 of these after the divergence between cattle and sheep.

### Transcription profiling by RT-PCR

**Figure 5:**
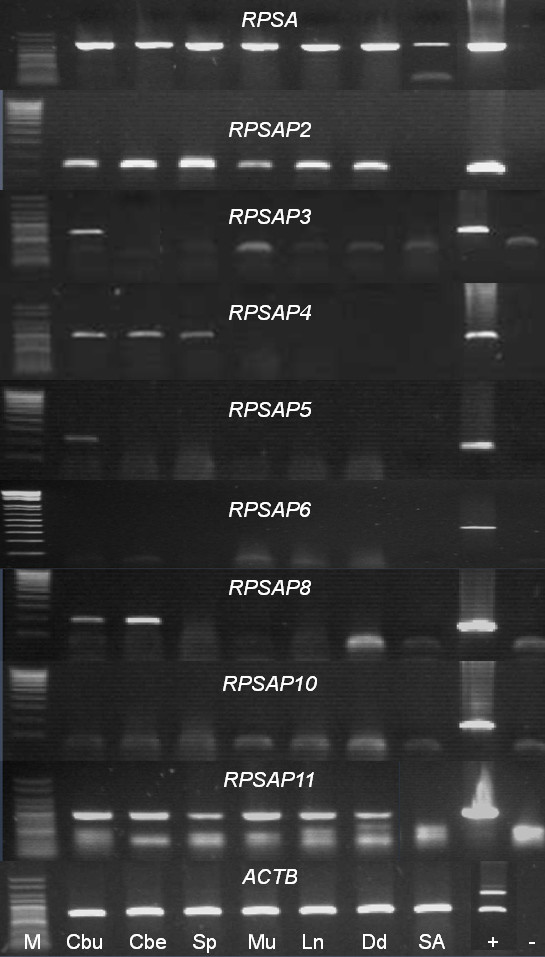
**Transcription profile of the *RPSA *gene family members**. Marker (M) is the Hyperladder V or IV (Bioline). Samples are cerebrum (Cbu), cerebellum (Cbe), spleen (Sp), muscle (Mu), lymph node (Ln), duodenum (Dd), blood (Bl) genomic or BAC DNA (+) and water (-).

To investigate whether some of the ovine *RPSA *pseudogenes were potentially functional, transcription profiling was performed by RT-PCR for all sheep *RPSA *family members in 7 tissues (Figure 5) i.e. cerebrum, cerebellum, spleen, muscle, lymph node, duodenum and blood. To be sure that no genomic DNA was present in the RNA samples, they were treated with DNase and checked by minus RT control PCR (Additional file 9). For 8 members of the *RPSA *family, gene-specific primers could be designed and their specificity was proven by checking that the primers did not amplify any other *RPSA *family member using the respective unique BAC clones as template (Additional file 10). Because *RPSA, RPSAP1, RPSAP7 *and *RPSAP9 *share a high level of sequence identity, no specific primers could be designed for these *RPSA *family members, they were tested with aspecific primers. All generated amplicons were sequenced. *RPSA *was expressed in all tested tissues. This agrees with the results of Marcos-Carcavilla et al. and Qiao et al. [18,33]. None of the pseudogenes was transcribed in blood. *RPSAP2 *and *RPSAP11 *were transcribed in all other tested tissues, while *RPSAP3*, *RPSAP5 *and *RPSAP8 *were only transcribed in one or more brain regions and *RPSAP4 *was transcribed in brain regions and spleen. *RPSAP6 *and *RPSAP10 *were not expressed in any of the tested tissues. In the case of *RPSAP1, RPSAP7 *and *RPSAP9*, tested with aspecific primers which all could also amplify *RPSA*, we generated amplicons which, after sequencing, turned out to be all *RPSA *transcripts. Thus we can conclude that *RPSAP1, RPSAP7 *and *RPSAP9 *are not expressed or at a very low level compared to the active *RPSA *gene. Therefore it would be interesting to do RT-qPCR with specific probes in order to be sure if the pseudogenes are expressed at very low levels or not at all. No clear relationship between the transcription profile of the various pseudogenes and the *in silico *prediction of possible promoters was observed. For instance, *RPSAP10 *is not expressed in any tissue tested although we did predict a promoter in the upstream sequence. Thus it may be possible that *RPSAP10 *is expressed in other tissues not examined in this study or that it has a low level of transcription. In addition, the *in silico *predicted promoter might not act as a *cis*-regulatory element *in vivo*. In contrast, *RPSAP3 *is transcribed in certain brain regions although we did not predict any promoter, probably because the promoter is located more upstream than the region analyzed here.

## Conclusions

In addition to the already described ovine *RPSA *gene, we have identified 11 members of the ovine *RPSA *gene family, and designated them *RPSAP1-RPSAP11 *since they are all considered to be processed pseudogenes. The flanking genomic regions of each *RPSA *family member was analyzed by annotating the constructed BAC contigs, which revealed 40 genes (of which 37 had not been previously described in sheep) based on comparative mapping. All these regions show conserved synteny with the orthologous bovine counterparts and the locations were confirmed by FISH. Five pseudogenes have a bovine counterpart. *In silico *analysis predicted the presence of 55 more *RPSA *pseudogenes in the bovine genome.

Compared to the *RPSA *transcript, *RPSA *pseudogenes differ significantly both in structure and sequence identity, ranging from structurally identical pseudogenes sharing 99% sequence identity to pseudogenes lacking half of the gene or containing many deletions throughout the whole gene, sharing only 74% sequence identity. A remarkable result is that at least 6 of the 11 pseudogenes are transcriptionally active. However, whether these transcripts are functional as regulatory non-coding RNA or as functional proteins remains to be investigated.

In previous studies, 1 to 3 *RPSA *pseudogenes per species, discovered while screening with the intention to isolate the full length functional *RPSA *gene, were characterized. Furthermore, the number of *RPSA *pseudogenes in 4 species with fully sequenced genomes was determined by genome-wide *in silico *screening but those pseudogenes were not characterized (Table 1). Here we report in detail the characterization of the *RPSA *gene family in a species. A strategy was developed to isolate all the ovine *RPSA *pseudogenes sharing a high level of sequence identity with *RPSA*. We screened with 8 different primers representing each exon at least once and with a Ta that was at least 8°C lower than the Tm. All BAC clones were positive for at least 2 primer pairs and there was no concordance between the number of BAC in a mini-contig and the level of sequence identity with *RPSA*. Therefore, we conclude that it is most likely that we have isolated all the ovine *RPSA *pseudogenes that could interfere with the functional *RPSA *gene in genetic studies. The discrepancy between the numbers of ovine *RPSA *pseudogenes found (11) and the numbers described in genome-wide screenings (45-61) might be explained by the low sequence identity of most pseudogenes found *in silico*. In *Bos taurus *for instance, 51 of the 60 pseudogenes share an overall nucleic acid identity with the bovine *RPSA *gene beneath 80% (Additional file 8). Due to our experimental design, pseudogenes with a low sequence identity were not isolated since it is not likely that those pseudogenes would interfere with molecular studies on the functional full length *RPSA *gene.

In conclusion, we describe 11 ovine processed *RPSA *pseudogenes. This knowledge on their structure and sequence will facilitate the molecular genetic studies of the functional gene since it will now be possible to take into account the existence of the pseudogenes in the design of such studies.

## Methods

### Construction BAC mini-contigs

The ovine INRA BAC library, consisting of 90.000 clones with an average insert length of 123 kb and a genome equivalent of 3.4, was screened by PCR [19]. The primers were designed using Primer3, based on conserved regions in the sheep *RPSA *gene [34]. The conserved regions were detected by comparison of all ovine ESTs available in GenBank that shared similarity with the published ovine mRNA sequence of *RPSA *[GenBank:EF649775] with BLAST and ClustalW [21,35]. PCR was conducted with Faststart Taq DNA Polymerase (Roche). PCR conditions were 5 min at 95°C, 40 cycles of 30 s at 95°C, 30 s at 50°C and 1 min at 72°C, and a final 10-min elongation step at 72°C. Thirty-nine superpools, each consisting of 44 pools (24 plates, 8 rows and 12 columns), were screened. Each positive combination was verified by colony PCR.

All isolated BACs were grown in a 200 ml culture from which DNA was purified with the Qiagen Plasmid Midi kit (Qiagen) according to the manufacturer's instructions. The BAC ends were sequenced with the universal primer (UP) (5'-CGACGTTGTAAAACGACGGCCAG-3') and reverse primer (RP) (5'-CACAGGAAACAGCTATGACCATGATTACG-3') primers with 1 μg of purified BAC DNA as template. Unique STS primer pairs (Additional file 2), based on the BESs, were used to screen all isolated BACs and to construct mini-contigs.

All sequencing was performed with the Big Dye Terminator mix (Applied Biosystems) and analyzed on an ABI-3730xl Analyser (Applied Biosystems).

### Characterization of RPSA gene family members

The primers used to screen the INRA BAC library were used as initial sequence primers to sequence the *RPSA *family member in one BAC of each mini-contig by direct sequencing. The obtained sequence was then used to develop new sequencing primers until the whole gene and an additional ± 500 bp upstream and ± 500 bp downstream of the sequence showing similarity with *RPSA*, was sequenced. If the screening primer did not work as sequencing primer, the amplicon generated with the screening primer was cloned into a pCR 2.1 vector with the TA Cloning Kit (Invitrogen) and the vector was transformed in DH5α Competent Cells (Invitrogen). The insert was then sequenced with UP and RP primers. All sequences were assembled into continuous sequences with CAP3 and analyzed with FGENESH and NCBI ORF Finder [20,36,37]. Promoter sequences were searched with CISTER, Neural Network Promoter Prediction, FPROM and TFsearch [36,38-40].

### Annotation of the mini-contigs by comparative mapping

All mini-contigs were annotated by comparing the BESs against bovine and human genomic sequences with NCBI BLAST [21]. Internal sequences were also annotated by PCR with primers based on bovine sequences of genes that were expected to be present in the mini-contig. All amplicons were verified and repeats were detected with Repeatmasker [26].

### FISH

Fluorescent in situ hybridization was performed at INRA in Jouy-en-Josas (France). To prepare the probes, BAC DNA was extracted according to standard protocols and purified with the S.N.A.P. K1900-01 Miniprep kit (Invitrogen). DNA was then nick-translated with biotin-14-dATP (BioNick 18247-015 labeling system, Invitrogen) and mixed with 100× total sonicated herring sperm DNA and 100× total sonicated sheep DNA. Subsequently, it was precipitated with ethanol, slightly dried and resuspended in hybridization buffer.

For R-banded sheep chromosomes, embryo fibroblast cell cultures were synchronized with an excess of thymidine and treated with 5-bromo-2'-deoxyuridine during the second half of S phase [41].

FISH, signal detection and R-banding were performed as previously described [42]. Briefly, labeled probes were denatured at 100°C for 10 min and pre-hybridized at 37°C for 30 to 60 min before hybridization to the chromosomes. Chromosome identification and band numbering followed the standard sheep ideogram reported in ISCNDB2000 [43].

### Transcription profiling

Fresh tissue samples were obtained from a commercial sheep slaughterhouse, frozen in liquid nitrogen immediately after slaughtering, crushed into powder and frozen at -80°C. Total RNA was isolated with the Aurum Total RNA Fatty and Fibrous Tissue kit (Bio-Rad) as described in the instruction manual. Subsequently, a minus RT-PCR was performed with *actin, beta *(*ACTB*) primers on 1 μl RNA to confirm the absence of any DNA contamination (Additional file 9) as previously described [44]. If DNA was still present in the sample, an additional DNase treatment with RQ1 RNase-free DNase (Promega) and a spin column purification with Microcon YM-100 (Millipore) were carried out.

The RNA concentration and purity of the samples were measured with the Nanodrop ND-1000 Spectrophotometer (Isogen) and the RNA quality was determined by evaluation of the 28S and 18S ribosomal bands on a 0.8% agarose gel.

Then, 0.2-1 μg RNA was converted into cDNA with iScript cDNA synthesis kit (Bio-Rad) using random and oligo dT primers. A confirmation PCR on 10× diluted cDNA with *ACTB *primers (giving a different amplicon length on gDNA and cDNA) was performed.

Specific primers, based on the aligned sequences of the different *RPSA *family members (Additional file 3) were designed for 8 members of the *RPSA *family and specificity was proven (see above).

Due to the high level of nucleotide sequence identity among *RPSA*, *RPSAP1*, *RPSAP7 *and *RPSAP9*, it was not possible to develop specific primers for these, but we were able to develop several primer pairs which amplified different combinations of 2 to 6 *RPSA *family members. One primer for instance amplifies *RPSA*, *RPSAP1*, *RPSAP7 *and *RPSAP11*; another *RPSA *and *RPSAP7 *and a third one *RPSA*, *RPSAP1*, *RPSAP5 RPSAP7 *and *RPSAP11*.

The obtained amplicons were sequenced to determine/confirm which (pseudo)gene was transcribed.

## Authors' contributions

AVDB carried out the BAC library screening, mini-contig building, sequencing of the genes, annotation by comparative mapping, the transcription profiling and drafted this manuscript. MVP participated in the design of the study, participated in the screening of the BAC library and provided experimental support. AMC participated in the screening of the BAC library. KH supervised the BAC library screening. HH and MB carried out the FISH mapping experiments. AVZ supervised the study. LJP participated in the study design and supervised the study. All authors read and approved the final manuscript.

## Supplementary Material

Additional file 1 **Primers used for screening of the INRA BAC library**. Characteristics of primers used for screening of the INRA BAC library: sequence, location, melting temperature and amplicon length. Click here for file

Additional file 2 **STS primers BES**. Characteristics of primers used for STS content mapping: sequence, annealing temperature and amplicon length. Click here for file

Additional file 3 **Alignment of the mRNA of the ovine *RPSA *gene with 11 *RPSA *pseudogenes**. The startcodon, stopcodon, poly-adenylation signal exon-exon junctions and interspersed repeats are highlighted in yellow. The primers used to screen the INRA BAC library are highlighted in green. Click here for file

Additional file 4 ***In silico *promoter prediction**. The *in silico *predicted promoter sequence is shown with the transcription start site highlighted in red. Furthermore, the score of the prediction, identification of the promoter and location of the transcription start site in the sequence is shown. Click here for file

Additional file 5 **Characteristics BES**. Characteristics of the 68 BES: BES length, orthologous sequences and genes, and repeats present in the BES. Click here for file

Additional file 6 **Primers used to annotate the mini-contigs**. Characteristics of primers used to annotate the mini-contig: sequence, annealing temperature, amplicon size and annotation information. Click here for file

Additional file 7 **FISH experiments**. Pictures of FISH experiments of the 11 *RPSA *pseudogenes. Click here for file

Additional file 8 **Bovine *RPSA *pseudogenes**. Characteristics of bovine *RPSA *pseudogenes predicted *in silico *by BLAST analysis of the bovine genome (reference assembly, based on Btau_4.0). In yellow are the pseudogenes that are annotated as 'similar to Ribosomal protein SA pseudogene'. The bovine pseudogene *RPSAP1 *is highlighted in blue and the pseudogenes that aren't annotated yet in white. Click here for file

Additional file 9 **minus-RT PCR control on RNA isolated from blood**. RT-PCR with *ACTB *primers. Marker (M) is the Hyperladder V (Bioline). Samples are RNA isolated from blood, genomic DNA and water (-). Click here for file

Additional file 10 **Specificity of the expression primer for *RPSAP6***. PCR with expression primer for *RPSAP6*. Marker (M) is the Hyperladder IV (Bioline). Samples are the respective unique BAC clones of all *RPSA *family members, genomic DNA and water (-). Click here for file
