# Supplementary material for: Characterization of the ovine ribosomal protein SA gene and its pseudogenes
Source: BMC Genomics. 2010 Mar 16;11:179. doi: 10.1186/1471-2164-11-179 (PMC2850357; doi:10.1186/1471-2164-11-179)
Supplement: Additional file 3 — Alignment of the mRNA of the ovine RPSA gene with 11 RPSA pseudogenes. The startcodon, stopcodon, poly-adenylation signal exon-exon junctions and interspersed repeats are highlighted in yellow. The primers used to screen the INRA BAC library are highlighted in green. [file 1471-2164-11-179-S3.PDF]

```
               ....|....| ....|....| ....|....| ....|....| ....|....| ....|....|
                   5         15         25         35         45         55
                                                        Exon 1-2
mRNA_RPSA      CCTTTCGGCG CTACCCAGAG AGGGGTCCAT ACGGCGTTGT CTTGAATTCC CATCGTAACT
RPSAP7         TTTTTCGGCG CTACCCAGAG AGGGGTCCAT ACGGCGTTGT CTTGAATTCC CATCGTAACT
RPSAP1         GCTTTCGGTG CTACCCAGAG AGGGGTACAT ACGGCGTTGT CCTGAATTCC CATCATAACT
RPSAP9         CCTTTCGGCG CTACCCAGAG AGGGGTCCAT ACGGCGTTGT CTTGAATTCC CATCGTAACT
RPSAP4         TCTTTCCATG CTACCCAGAC AGGGGTCCAT ACAGAATTAT TCTAGATTCC CCTCGTAACT
RPSAP3         TTTTTTCACA CTACTAAGAC AGGGATCCAT ATGGCGTT-- -TTGGATTCC CACTGTAATG
RPSAP2         CTTTTATACA CTGCCCAGAG AAAGATCCAT ACAGCAATGT TCGGGGTTCC CATTTTAATT
RPSAP5         GCTTTCGGAG CTACCCAGAG AGGGGTCCAT ACGGCGTTGT CCTGAATTCC CATCGTAACT
RPSAP6         CCTCTCCTCG CTACCCAGAC TGGGGTCCAT ATGGCACTGT TCTGGATTCC CATTGTAACT
RPSAP11        .......... ......AGAG AGAGATTCAT ATGG-GTTGT TCTGGTTTCT CATCATAATT
RPSAP8         .......... .......... .......... .......... .......... ..........
RPSAP10        .......... .......... .......... .......... .......... ..........


               ....|....| ....|....| ....|....| ....|....| ....|....| ....|....|
                   65         75         85         95        105        115
                                                 OariRPSA+1/OariRPSA+4

mRNA_RPSA      TAAAGGGAAG CTTTCACAAT GTC------C GGAGCCCTTG ATGTCCTGCA AATGAAGGAG
RPSAP7         TAAAGGGAAG CTTTCACAAT GTC------C GGAGCCCTTG ATGTCCTGCA AATGAAGGAG
RPSAP1         TAA-----AG CTTTCACAAT GTC------C GGAGCCCTTG ATGTCCTGCA AATGAAGGAG
RPSAP9         TAAAGGGAAG CTTTCACAAT GTC------C GGAGCCCTTG ATGTCCTGCA AATGAAGGAG
RPSAP4         TAAAGGGGA- ---------- ---------- GGAGTCCTTG ATGTCTTGCA AATGAAGGAA
RPSAP3         TAAACGGAAA CTTTCACAAT GTC------T GCTGTCCTTG ATGTCCTGCA AATGGAGGAG
RPSAP2         TAAAGGAAAG CTTTCACAAT GTC------T AGAGTCCTTT ATGTCCTGCA GATGAAGGGG
RPSAP5         TAAAGGAAAG CTTTCACAAT GTC-SINE-C GGAACCCTTG ATGTCCTGCA AATGAAGGAG
RPSAP6         TAAAGGGAAA CTTTCACCAT ATC------C AGAGCCCTTG ATGTCTTGCA AAT-AAGGAG
RPSAP11        TAAAGAAAAA TACTCACAAT ATC------C AGAGCCCTTT ATGTCCTGCA AAAGAAAGAA
RPSAP8         .......... .......... .......... .......... .......... ..........
RPSAP10        .......... .......... .......... .......... .......... ..........


               ....|....| ....|....| ....|....| ....|....| ....|....| ....|....|
                  125        135        145        155        165        175
mRNA_RPSA      GAG-----GA TGTCCTCAAA TTCCTT---- ------GCAG CAGGAACCCA CTT------A
RPSAP7         GAG-----GA TGTCCTCAAA TTCCTT---- ------GCAG CAGGAACCCA CTT------A
RPSAP1         GA-------- TGTCCTCAAA TTCCTT---- ------GCAG CAGGAACCCA CTT------A
RPSAP9         GAG-----GA TGTCCTCAAA TTCCTT---- ------GCAG CAGGAACCCA CTT------A
RPSAP4         CAG-----GA TGTCCTCAGA TTCCTT---- ------GCAG CAGGAACCCA CTC------A
RPSAP3         GGT-----GG TGTCCTCAAA TTCCTT---- ------GCAG TGGGAACCCA CGT------A
RPSAP2         GTG-----GG TGTCCTTAAA TTTCTT---- ------GCAG CTGGAACCCA CT--------
RPSAP5         GAG-----GA TGTCCTCAAA TTCCTT---- ------ACAG CAGGAACCCA CTT------A
RPSAP6         GAGCCCTTGA TGTCCTCAAA TTCCTT---- ------TCAT CAGGAACTCA CTT-SINE-A
RPSAP11        GAA-----GA GGTCCTCAAA TTCCTT-tRNA-SINE-ACAT AAGGAACCCA TTT------A
RPSAP8         .......... .......... .......... .......... .......... ..........
RPSAP10        .......... .......... .......... .......... .......... ..........
```

```
               ....|....| ....|....| ....|....| ....|....| ....|....| ....|....|
                    185        195        205        215        225        235
                        OariRPSA+6
                                                                       Exon 2-3
mRNA_RPSA      GGTGGCACCA ACCTTGACTT CCAAATGGAA CAGTACATCT ACAAAAGGAA AA-GTGATGG
RPSAP7         GGTGGCACCA ACCTTGGCTT CCAAATGGAA CAGTACATCT ACAAAAGGAA AA-GTGATGG
RPSAP1         GGTGGCATCA ACCTTGACTT CCAAATGGAA CAGTACATCT ACAAAAGGAA AA-GCGATGG
RPSAP9         GGTGGCACCA ACCTTGACTT CCAAATGGAA CAGTACATCT ACAAAAGGAA AA-GTGATGG
RPSAP4         TGTGGCACCT ACCTTGACTC CCAAAGGGAA CAGTGCGTCT ACAAAAGGGA GC-ATGGTGG
RPSAP3         GGTGGCACCA ACCTTGACTT CCAAATGGAA CAGTACACCT ACAGAAGGAA AA-GTTATGG
RPSAP2         ---------- --CTTGACTT CCAAATGAAA CGGTACATCT ACAAAAGGAA AA-ATGATGG
RPSAP5         GGTGGCACCA ACCTTGACTT CCAAATGGAA CAGTACATCT ACAAAAGGAA AA-GTGATGG
RPSAP6         GGTGGCACCA ACTTTAACTT TCAAATGAAA CAGCATGTCT ACAAAAGGAA AA-GT-----
RPSAP11        GGAGGCACTT AGGTTGACTT CCAAATGGAA CAGTGAATCT ACAAAAGGAA AAAGTGATGG
RPSAP8         .......... .......... .......... .......... .......... ..........
RPSAP10        .......... .......... .......... .......... .......... ..........

               ....|....| ....|....| ....|....| ....|....| ....|....| ....|....|
                    245        255        265        275        285        295
                         OariRPSA-1

mRNA_RPSA      CATCTACATC ATAAACCTGA AGAGGACCTG GGAGAAGCTG CTGTTGGCCG CTCGGGCCAT
RPSAP7         CATCTACATC ATAAACCTGA AGAGGACCTG GGAGAAGCTG CTGTTGGCCG CTCGGGCCAT
RPSAP1         CATCTACATC ATAAATCTAA AGAGGACCTG GGAGAAGCTT CTGTTGGCCG CTCGGGCCAT
RPSAP9         CATCTACATC ATAAACCTGA AGAGGACCTG GGAGAAGCTG CTGTTGGCCG CTCGGGCCAT
RPSAP4         CATCTCCATC ATGAATCCGA AGAGAACCTG GGGGAAGCTT CTGTTGGCAG TTTGTGCCAT
RPSAP3         CGTCTACATC ATAAATCTGA AGAGAACTTG GGAGAAGCTT CTGTTGGCAG CTTGTGCCAT
RPSAP2         CATCTACATC ATAAATCTAA A---ACCCTG GGAGGAGCTT CTGTTGGCAG GTCGTGCGAT
RPSAP5         CATCTACATC ATAAATCTGA AGAGGACCTG GGAGAAGCTT CTGTTGGTAG CTCCGGCCAT
RPSAP6         ---------- ------CTGA AGAGAACCTT GCAGAAGCTT CTGTTGGCAG CTCATGCCAT
RPSAP11        TGTCTACCTC ATAAATCTGA AGAGAATGTG GGAGAAAGTT CTG---GCAG CTTGTGCCAT
RPSAP8         .......... .......... .......... .......... .......... ..........
RPSAP10        .......... .......... .......... .......... .......... ..........

               ....|....| ....|....| ....|....| ....|....| ....|....| ....|....|
                    305        315        325        335        345        355
                                                    OariRPSA+5

mRNA_RPSA      -----TGTCG CCATTGAGAA CCCGGCGGAC GTCAGTGTCA TCTCCTCCAG GAACACTGGC
RPSAP7         -----TGTCG CCATTGAGAA CCCGGCGGAC GTCAGTGTCA TCTCCTCCAG GAACACTGGC
RPSAP1         -----TGTCC CCATTGAAAA CCCGGCTGAT GTCAGTGTCA TATCCTCTAG GAATACTGAC
RPSAP9         -----TGTCG CCATTGAGAA CCCGGCGGAC GTCAGTGTCA TCTCCTCCAG GAACACTGGC
RPSAP4         -----TTTTG CCGCTGAAGG TCCAGCTGAT GCCGG-GTCA TATCCTCCAG GA--------
RPSAP3         -----TGTTG CCTTTGAAAA CCCAGCTGAT GTCAGTGTCA CATCCTCCAG GAATACTAGC
RPSAP2         -----TGTTG CCATTGAAAG CCCAGCTGAT GTGAGTGTCC CATCCTCCAG GAA-------
RPSAP5         -----TGTTG CCATTGAAAA CCCGGCTGAT GTCAGTGTCA TATCCTCCAG GAATACTGGC
RPSAP6         -----CGTTG TCATTGAAAG CCCAGTGGAC ATCAGCGTCA TTTCCTCCGA GAATACTGGC
RPSAP11        GCCATTGATC CCACTGAAAA CTCGGCTAAT GTCAGTGTCC TGTCCTCCAG ATATACTGGC
RPSAP8         .......... .......... .......... .......... .......... ..........
RPSAP10        .......... .......... .......... .......... .......... ..........
```

```
                         ....|....| ....|....| ....|....| ....|....| ....|....| ....|....|
                            365        375        385        395        405        415
                           OaLam4+

                         Exon 3-4
mRNA_RPSA                CAGCGAGCTG TGCTGAAGTT TGCTGCTGCC ACTGGAGCCA -CTCCTATCG CTGGCCGCTT
RPSAP7                   CAGAGAGCTG TGCTGAAGTT TGCTGCTGCC ACTGGAGCCA -CTCCTATCG CTGGCCGCTT
RPSAP1                   CAGTGAGCTG TGCTGAAGTT TGCTGCTGCC ACTGGAGCCA -CTCCTATCG CTGGCCGCTC
RPSAP9                   CAGAGAGCTG TGCTGAAGTT TGCTGCTGCC ACTGGAGCCA -CTCCTATCG CTGGCCGCTT
RPSAP4                   ---------- ---------- TGCTGCTGCC GCGGGAGCTA -CTCCTTTTG CTGGCTGCTT
RPSAP3                   CAGTGAGCTA TGCTGAAGT- TGTTGCTGCC ACTGGAGCTA TTTCCTATTA CAGGCCACTT
RPSAP2                   ------GCTG TGCGGAAGCT TGCTGCTGCT CCTGGAGCCG -CTCCTATTG CTGGGCGCTT
RPSAP5                   CAGCGAGCTG TGCTGAAGGT TGCTGCTCCC ACTGGAGCCA -CTCCTGTTG CTGGCTGCTT
RPSAP6                   CTGCGGGCTG TGCTGAAGTT TGCTGCTGCC ACTGGAGCCA -TTCCTATTG CTGGCTGCTT
RPSAP11                  CACCAAGCTG TGCTGAAGTT TGCTGCTGCC CCAAG-GCCA T-TCCTGTTG CTGACTGTGT
RPSAP8                   .......... .......... .......... .......... .......... ..........
RPSAP10                  .......... .......... .......... .......... .......... ..........


                         ....|....| ....|....| ....|....| ....|....| ....|....| ....|....|
                            425        435        445        455        465        475
                                OariRPSA+2/OariRPSA-4

mRNA_RPSA                CACTCC-GGG AACCTTCACT AACCAGATCC AGGCCGCCTT CAGGGAGCCG AGGCTTCTGG
RPSAP7                   CACTCC-GGG AACCTTCACT AACCAGATCC AGGCCGCCTT CAGGGAGCCG AGGCTTCTGG
RPSAP1                   CACTCC-GGG AACCTTCATT AACCAGATCC AGGCCGCCTT CAGGGAGCCG AGGCTTCTGG
RPSAP9                   CACTCC-GGG AACCTTCACT AACCAGATCC AGGCCGCCTT CAGGGAGCCG AGGCTTCTGG
RPSAP4                   CACTCC-CAG AACCTTCACT AACCAGATTC AGACAGCCTA CCAGGAGCCA AGACTTCTGG
RPSAP3                   CAGTCCTGGG AACTTTCACT AACCAGATCC AGGCAGCCTT CCGGGAGCCA AGACTTCAGT
RPSAP2                   CTCTCCT-GG A-CCCTCACT AACCAGATCC GGGCAGCCTT CCAGGAGCCA CAATTTCTGG
RPSAP5                   CACTCC-GGA AGCCTTCACT AACCAGATCC AGGCCGCGTT CAGGGAGCCA AGGCTTCTAG
RPSAP6                   CACTCT-TGG AACCTTCGCT AACCAGAACT AGGCAGCCTT CCAGGAGCCA AGGCTTCTGC
RPSAP11                  CAGTCCT-GG AGATTTCACT CACCAGATCC AGGCAGTCTT CTGGGAGTGG GGACTTCCGG
RPSAP8                   .......... .......... .......... .......... .......... ..........
RPSAP10                  .......... .......... .......... .......... .......... ..........


                         ....|....| ....|....| ....|....| ....|....| ....|....| ....|....|
                            485        495        505        515        525        535
mRNA_RPSA                TGGTCACCGA TCCCAGGGCT GACCACCAGC CCCTCACGGA AGCCTCCT-A CGTTAACCTG
RPSAP7                   TGGTCACCGA TCCCAGGGCT GACCACCAGC CCCTCACAGA AGCCTCCT-A CGTTAACCTG
RPSAP1                   TGGTCACCGA TCCCAGGGCT GACCACCAGC CCCTCACGGA AGCTTCCT-A CGTTAACCTG
RPSAP9                   TGGTCACCGA TCCCAGGGCT GACCACCAGC CCCTCACGGA AGCCTCCT-A CGTTAACCTG
RPSAP4                   CAGTTACTGA TCCCAGGGCT GTCCACCAGC CTCTCACAGA AACCTCCT-A TGTTAGCCTG
RPSAP3                   TAGTTACTGA TCCCAGGGCT GACCACCAGC CTCTCACAGA TGCCTCCT-A CATTAACCTG
RPSAP2                   TGGTTAC--- ---------- -------AGC TGCTCACAGA GACCTCTT-A CGTTCACCTG
RPSAP5                   CGGTTGCCAA TCCCAGGGCT GACCACCAAC CGCTCACAGA AGCCTCTTAA CGTTAACCTG
RPSAP6                   TCGTTACGAA TCGCAGGGCA GACCACCAGC CTCTCACAGA AGTCTC---- --TTCACCTG
RPSAP11                  TGGTTGCTGA TCCTAGGGCC ACGCACTAGC CTCTCACAGA GGCCTCTT-A CGTTAACCTG
RPSAP8                   .......... .......... .......... .......... .......... ..........
RPSAP10                  .......... .......... .......... .......... .......... ..........
```

```
              ....|....| ....|....| ....|....| ....|....| ....|....| ....|....|
                   545        555        565        575        585        595
mRNA_RPSA     CCAACCATTG CCCTGTGCAA CACGGACTCT CCTCTGCGCT ACG--TGGAC ATCGCCATCC
RPSAP7        CCAACCATTG CCCTGTGCAA CACGGACTCT CCTCTGCGCT ACG--TGGAC ATCGCCATCC
RPSAP1        CCAACCATTG CTCTGTGCAA CACGGACTCT CCTCTGCGCT ACG--TGGAC ATCGCCATCC
RPSAP9        CCAACCATTG CCCTGTGCAA CACGGACTCT CCTCTGCGCT ACG--TGGAC ATCGCCATCC
RPSAP4        CCTACC-TAC TACTGTGTAA CTCCGAT--- ---------- ---------- ----------
RPSAP3        CCTACCATTG CTCTGTGTAA CA--GACTCT CTTCTGTGTT ATG--CGGAC ATTGCCATCC
RPSAP2        CCCACCATTG CTCTGTGTAA CACAGACTCT GCAGAGAGGA GAGAGTGGAC GTTGTCATCC
RPSAP5        CCAACCACTG CTCTGTGTAA CACAGACTCT CCTCTGTGCT ACG--TGTAC ATTGCCATCC
RPSAP6        CCTTCCATTG ATCTATGTGA CACAGACTCT CCTCTCTGCT CCA--TGGCC CCAGCCATCG
RPSAP11       CCTACCACTG CTCTGTGTAA CTCAGACTCA ACTCCGCTCT ----GTGGGC ATTGCCATTT
RPSAP8        .......... .......... .......... .......... .......... ..........
RPSAP10       .......... .......... .......... .......... .......... ..........


              ....|....| ....|....| ....|....| ....|....| ....|....| ....|....|
                   605        615        625        635        645        655
                                                                  OariRPSA-2
                             Exon 4-5
mRNA_RPSA     CGTGCAA--- --CAACAAGG GGGCGCACTC AGTGGGCCTG ATGTGGTGGA ---TGCTCGC
RPSAP7        CGTGCAA--- --CAACAAGG GGGCGCACTC AGTGGGCCTG ATGTGGTGGA ---TGCTCGC
RPSAP1        CGTGCAA--- --CAACAAGG GAGCGCACTC AGTGGGTCTG ATGTG---GA ---TGCTCGC
RPSAP9        CGTGCAA--- --CAACAAG. .......... .......... .......... ..........
RPSAP4        ---------- -------AGG GAATGCCCTC AGTGGGGCTG ATGTGGTGGG ---TGCTCGC
RPSAP3        --TGCAA-SINE-CAACAAGG GAGCTCGCTC AGTGGGCCTG ATGTGGTGGT GGTTGCAGAC
RPSAP2        CATGCAA--- --CAAGAA-- -GGCTGACTT GGTGGGTCTG ATGTGGTGGA ---TACTCAC
RPSAP5        CATTCAA--- --CAACAAGG GAGCACACTC ATGTGCGCTG ATGTTGTGGA ---TGCTCCC
RPSAP6        CGTG------ --TGACAATG GAGCTCACTC AGTGGGTCTG ACGTGGTGGG ---TGCTCGC
RPSAP11       CCTCCAA--- --CAACAAGG GAGCTCACTC AGTGGGTCTG ---TGACGGG ---TGCTGGC
RPSAP8        .......... ......AAGG GCGTGCACCC AGCGGGTCTG ACGTGGTGGA ---TGCGCGC
RPSAP10       .......... .........G GAGCGCACTC AGTGGGTCTG ATGTGGTGGC ---TGCTCGC


              ....|....| ....|....| ....|....| ....|....| ....|....| ....|....|
                   665        675        685        695        705        715
mRNA_RPSA     CCGGGAA-GT CCTGCGCATG CGCGGCACCA TCTCCCGAGA ACACCCGTGG GAGGTCATGC
RPSAP7        CCGGGAA-GT CCTGCGCATG CGCGGCACCA TCTCCCGAGA ACACCCGTGG GAGGTCATGC
RPSAP1        CTGGGAA-GT CCTGCGCATG CGTGGCACCG TCTCCCGAGA ACACCCGTGG GAGGTCATGC
RPSAP9        .......... .......... .......... .......... .......... ..........
RPSAP4        CTGGGAA-GT TCTGCACATG TGTGGCACCA TCTCTCATGA GCACCCACAG AAGGTCATGC
RPSAP3        CTGCGAA-TT TCTGTGCATG TGTGGCATCA TCTCCTGGGA ACACCCATGG GAAGTCATGC
RPSAP2        CTGGGGAAGT TCTGCGCCTA CATGGCGCCA TCTCCTGTGA ACGCCGATGG GAGGTCATGG
RPSAP5        CCGGGAA-GT TCTGTGCATG AGTGGCACCA TCTCCTGAGA ACACCCGTGG GAGGTCATGC
RPSAP6        CAGAGAA-GT TCTGCGAATG CATGACACCA TCTCCTGTGA ACACC-CTGG AAGGTCATGC
RPSAP11       CTACGAA-GT TGTACACATG CATGGCACCA TCTCTCATGA ACACCCACAG GAGGTCACAC
RPSAP8        CCGGGAA-GT TCTGCACATG CGTGGCACCA TCTCCCGAGA ACGCCCATGG GAGGTCATGC
RPSAP10       CCGGGAA-AT CCTGCGCATG CGTGGCACCA TCTCCCGAGA ACACCCGTGG GAGGTCATGC
```

```
             ....|....| ....|....| ....|....| ....|....| ....|....| ....|....|
                   725        735        745        755        765        775
                                                              OariRPSA-5
                                                Exon 5-6
mRNA_RPSA    CGGACCTCTA CTTCTACAGG GACCCCGA-G GAGATTGAAA AGGAAGAGCA GGCAGCAGCC
RPSAP7       CGGACCTCTA CTTCTACAGG GACCCCGA-G GAGATTGAAA AGGAAGAGCA GGCAGCAGCC
RPSAP1       CGGACCTCTA CTTCTACAGA GATCCTGA-G GAAATTGATA AGGAAGAGCA GGCAGCAGCT
RPSAP9       .......... .......... .......... .......... .......... ..........
RPSAP4       CTGATCTCTG CTTCTACAGA GATCCTGA-A GAGATTGAAA GGGAAGAGCA GACGGCAGCT
RPSAP3       CTGATCTCTG CTTCTACAGA GGTCCTGA-A GAGTTTGAAA AGGAATAATA GGTGGCAGCT
RPSAP2       CTGA------ -----ACAGA GACCCTGA-A GAGATTGAAA AAGAAGAGCG GGCAGCAGCT
RPSAP5       CGGACCTCTG CTTCTACAGA GAGCCTGA-A GAGATTGAAA AGGAAGAGCA GGCAGCAGCT
RPSAP6       CCCATCTTTC CTTCTATGGA GATGCTGACA GGGATTGAAA AGGAAGAGCG GGTGGCAGCT
RPSAP11      CTGATCTCAG CGTCCACACA GACCCAGA-A GAGATTGAAG AGGAGGAGCA GGCTGCTGCT
RPSAP8       CGGATCTCTA CGTCTACAGA GATCCTGG-A GAGCCTGAAA AGGAAGAGCA GGCAGCCGCT
RPSAP10      CGGACCTCTA CTTCTACAGA GATCCTGA-G GAGATTGAAA AGGAAGAGCA GGCAGCAGCT


             ....|....| ....|....| ....|....| ....|....| ....|....| ....|....|
                   785        795        805        815        825        835
                                   OariRPSA+3

mRNA_RPSA    GAGAAGGCTG TGACCAAGGA GGAGTTTCAG GGCGAATGGA CTGCTC---- ----------
RPSAP7       GAGAAGGCTG TGACCAAGGA GGAGTTTCAG GGCGAATGGA CCGCTC---- ----------
RPSAP1       GAGAAGGCTG TGACCAAGGA GGAGTTTCAG GGTGAATGGA CCGCTC---- ----------
RPSAP9       .......... .......... .......... .......... .......... ..........
RPSAP4       GAGAAGGCAG TGACCGATGA GGAGTTTCAG AGTTAATGCA CCGCTC---- ----------
RPSAP3       GAGAAGGCTG TGACCAAGGA GGAATTTCAG AGTGAATGGA CTGCTC-19bpINSERTION-
RPSAP2       GAGAGG-TTG TGACCAAGGA GGAATTTTAC GGTTACTAGA CCCATT---- ----------
RPSAP5       GAGAAGGCTG TGACCAAGGA GGAATTTCAG GGTGAATGGA CTGCTC---- ----------
RPSAP6       AAGAAGGTTG TGACCAAGGA GGCATTTCAG GGTGAATGGA CCGCTC---- ----------
RPSAP11      CAAAAGGCTG TGACCAAAGA GGCATTTCAG TGTGAGTGGA CAGCTC---- ----------
RPSAP8       GAGAAGGCTG TGGCCATGGA GGAGTTTCAG GGTGAATGGA CTGCTC---- ----------
RPSAP10      GAGAAGGCTG TGACCAAGGA GGAGTTTCAG GGAGACTGAA CCACTC---- ----------


             ....|....| ....|....| ....|....| ....|....| ....|....| ....|....|
                   845        855        865        875        885        895
mRNA_RPSA    CAGCTC-CAG AGTTCACGGC TGCCCAGCCT GAGGTGGCAG ACTGGTCTGA AGGTGTGCAG
RPSAP7       CAGCTC-CAG AGTTCACGGC TGCCCAGCCT GAGGTGGCAG ACTGGTCCGA AGGTGTGCAG
RPSAP1       CAGCTC-CAG AGTTCACGGC TGCCCAGCCT GAGGTGGCAG ACTGGCCCAA AGGCGTGCAG
RPSAP9       .......... .......... .......... .......... .......... ..........
RPSAP4       CAGCTC---- ---TGAGGTG TACTCAGTCT GAGGTAGCAG ATTGGTCTGA AGGCAGGCAG
RPSAP3       TTGCTCACTG AGTTCACTGC TACTCAAACT GAGG-GCTGA ACTGGTCCAA AGGCATGCAG
RPSAP2       CAGCTC-CTA AGTTCTTTGC TGCTTGACCT GAGGCGGCAG ACTGGTCTGC AGGCGTGCAG
RPSAP5       CAGCTC-CAG AGTTCACTGC TACTCAGCCT GAGGTGACAG ACTGGTCCGA AGGTGTGCAA
RPSAP6       CAGCTC-CTG AGTTCACCGA TGCTCAACCT GAGGTGGTGG .......... ..........
RPSAP11      TAGCTC-CTG AGTT-ACTGC TGCTCAACCT GAGATCCCAG ACTGGAGTGC A-GCGTACAG
RPSAP8       CAGCTC-CAG AGTTCACTGC TACTCAGCCT GAGGTGGCAG ACTGGTCTGA AGGTG-----
RPSAP10      CAGCTC-CAG AGTTCACGGC TGCCCAGCTT GAGGTGGCAG ACTGGTCCGA AGGCGTGCAG
```

```
                   ....|....| ....|....| ....|....| ....|....| ....|....| ....|....|
                       905       915       925       935       945       955
                         OariRPSA+8                      OariRPSA-6/OariRPSA-3
                                                Exon 6-7
mRNA_RPSA          GTGCCTTCCG TGCCCATTCA GCAGTTCCCC ACTGAAGACT GGAGTGCTCA GCCTTCCACT
RPSAP7             GTGCCTTCCG TGCCCATTCA GCAGTTCCCC ACTGAAGACT GGAGTGCTCA GCCTTCCACT
RPSAP1             GTGCCTTCCG TGCCCATTCA GCAGTTCCCC ACTGAAGACT GGAGTGCTCA GCCTTCCACT
RPSAP9             .......... .......... .......... .......... .......... ..........
RPSAP4             TGCTCTCTGT GCC....... .......... .......... .......... ..........
RPSAP3             GAAC--TCAG C----TCTCA GCGGTTCCCC ACTGAAGACT GGAGTGCTCA GCCTGCCACT
RPSAP2             GTGCCGTTGG TGCCTATTTG GCAGCTCCCT GCTGCAGCCT GGAGTGCTGA GCCTGCCACT
RPSAP5             GTGCCTTCCG TGCCTATTCA GCAGTTCCCT GCTGAAGACT GGAGTGCTCA GCCTTCCACT
RPSAP6             .......... .......... .......... .......... .......... ..........
RPSAP11            GTGCCCTGGG TGCCTACTCA GCAG-TCCCT CCTGAAGACT GGAGTACGCA G-CTCCCACT
RPSAP8             ---------- --CCTATTCA GCAGTTCCCC ACTGAAGCCT GGAGTGATCT GCCTTCCACT
RPSAP10            GTGCCTTCCA TGCCCATTCA GCAGTTCCCT ACTGAAGACT GGAGTGCTCA GCCTTCCACT


                   ....|....| ....|....| ....|....| ....|....| ....|....| ....|....|
                       965       975       985       995       1005      1015
                                                                     OariRPSA-8

mRNA_RPSA          GAAGACTGGT CTGCAGCCCC CACTGCCC-A GGCCACGGAA TGGGTAGGAA CCACCACCGA
RPSAP7             GAAGACTGGT CTGCAGCCCC CACTGCCC-A GGCCACGGAA TGGGTAGGAA CCACCACCGA
RPSAP1             GAAGACTGGT CTGCAGCCCC CACTGCCC-A GGCCACGGAA TGGGTAGGAA CCACCACCGA
RPSAP9             .......... .......... .......... .......... .......... ..........
RPSAP4             .......... .......... .......... .......... .......... ..........
RPSAP3             GCACACTGGT CTGTAGCTCC CACCTGTC-A AGCCACTGAA TGGGTAGAAG CAACCACTGA
RPSAP2             GAAGACCGCT GTGC-CCCTC CACTGGTC-- AGCCCCTGAA GATGAGGGAG CGACCCCTGA
RPSAP5             GAAGACTGGT CTGCAGCTCC CACTGCTC-A GGCCACTGAA TGGGTCGGAA CCACCACTGA
RPSAP6             .......... .......... .......... .......... .......... ..........
RPSAP11            G......... .......... .......... .......... .......... ..........
RPSAP8             GAAGACTGGT CTGCAGCTCC CAAGGCTC-A GGCCACTGAA TGGGTAGGAA CCACCACTGA
RPSAP10            GAAGACTGGT CTGCAGCCCC CACTGCCCGA GGCCACGGAA TGGGTAGGAA CCACCACAGA


                   ....|....| ....|....| ....|....| ....|....| ....|....| ....|....|
                       1025      1035      1045      1055      1065      1075
mRNA_RPSA          GTGGTCGTAA GCTCTTCTTC CAAACACTTG TAGAACTTGC ACCAAAATGG AAATCTGGTT
RPSAP7             GTGGTCGTAA GCTCTTCTTC CAAACACTTG TAGAACTTGC ACCAAAATGG AAATCTG...
RPSAP1             GTGGTCCTAA GTTGTTCTT. .......... .......... .......... ..........
RPSAP9             .......... .......... .......... .......... .......... ..........
RPSAP4             .......... .......... .......... .......... .......... ..........
RPSAP3             GTGGTCTTAA GTTGTTCTTC CACAAACTTT .......... .......... ..........
RPSAP2             GGAGTCTTAA GCTGTTCTTC CA........ .......... .......... ..........
RPSAP5             GTGGTTGTAA GCAGTTCTTC CAAACACTTG TAGAACTTCC ACAAA..... ..........
RPSAP6             .......... .......... .......... .......... .......... ..........
RPSAP11            .......... .......... .......... .......... .......... ..........
RPSAP8             GTGGTCATAA GCTGTTCTTC CAAACACTTG TAGAACTTCC ACAAA..... ..........
RPSAP10            GTGGTCGTAA GCTGTTCTTC CAAACACCTG TAGAACTTGC ACCAAAATGG AAATCTG...


                   ....|....| ....|....|
                       1085      1095
mRNA_RPSA          GATGGAAATA AACTGTTTCT
```
