# Supplementary material for: Characterization of the ovine ribosomal protein SA gene and its pseudogenes
Source: BMC Genomics. 2010 Mar 16;11:179. doi: 10.1186/1471-2164-11-179 (PMC2850357; doi:10.1186/1471-2164-11-179)
Supplement: Additional file 7 — FISH experiments. Pictures of FISH experiments of the 11 RPSA pseudogenes. [file 1471-2164-11-179-S7.PDF]

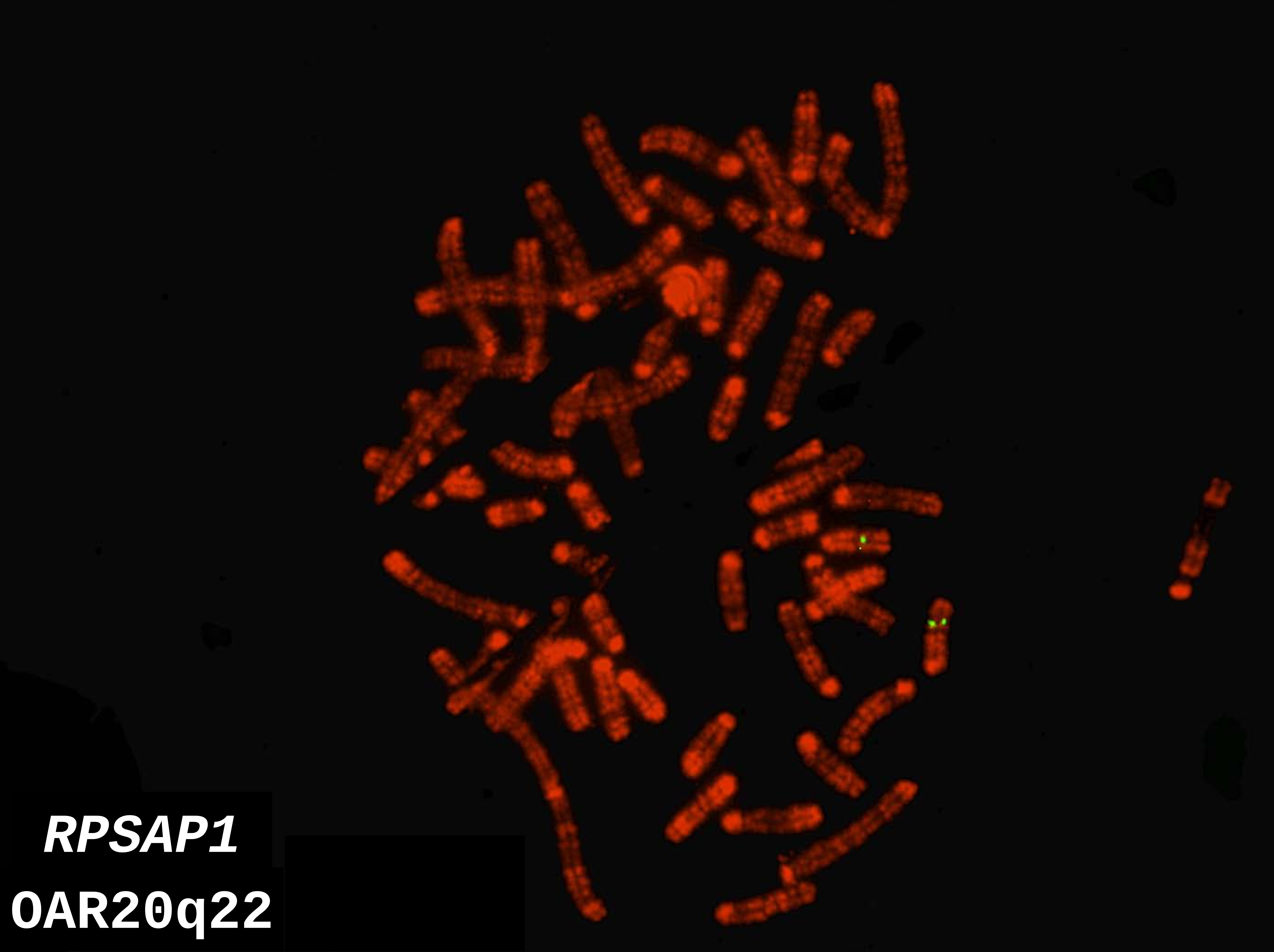
RPSAP1
OAR20q22

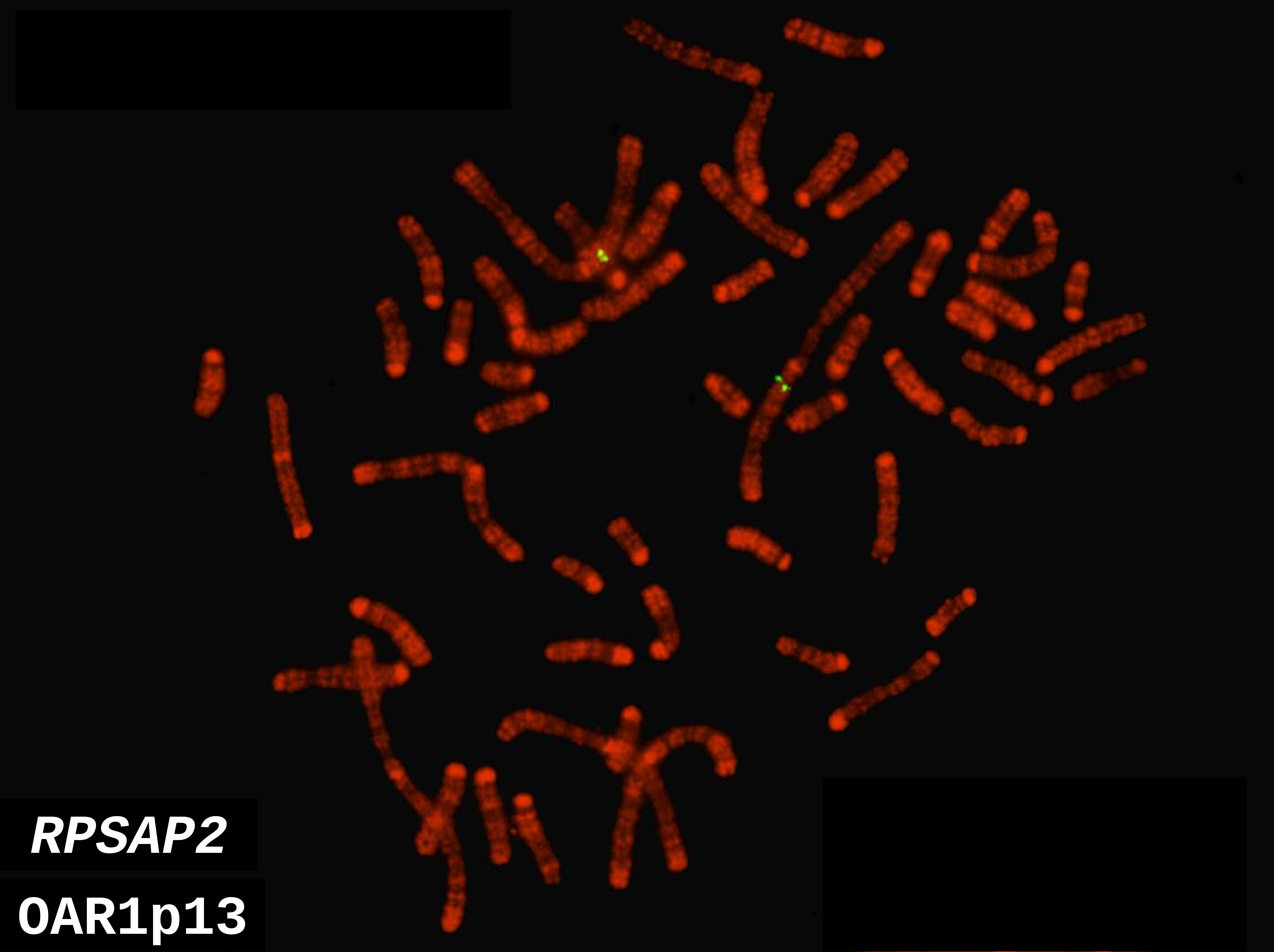
RPSAP2
OAR1p13

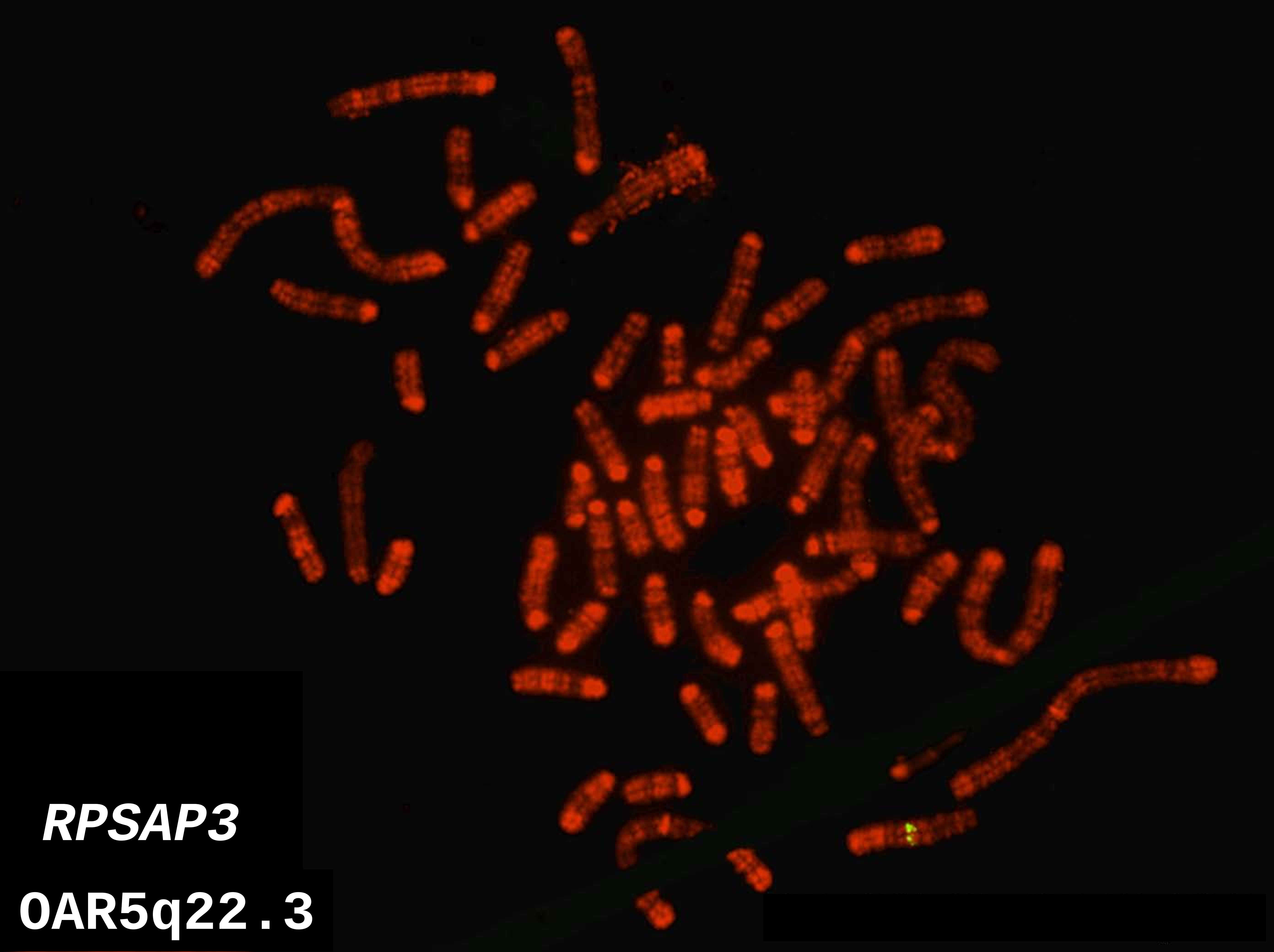
RPSAP3
OAR5q22.3

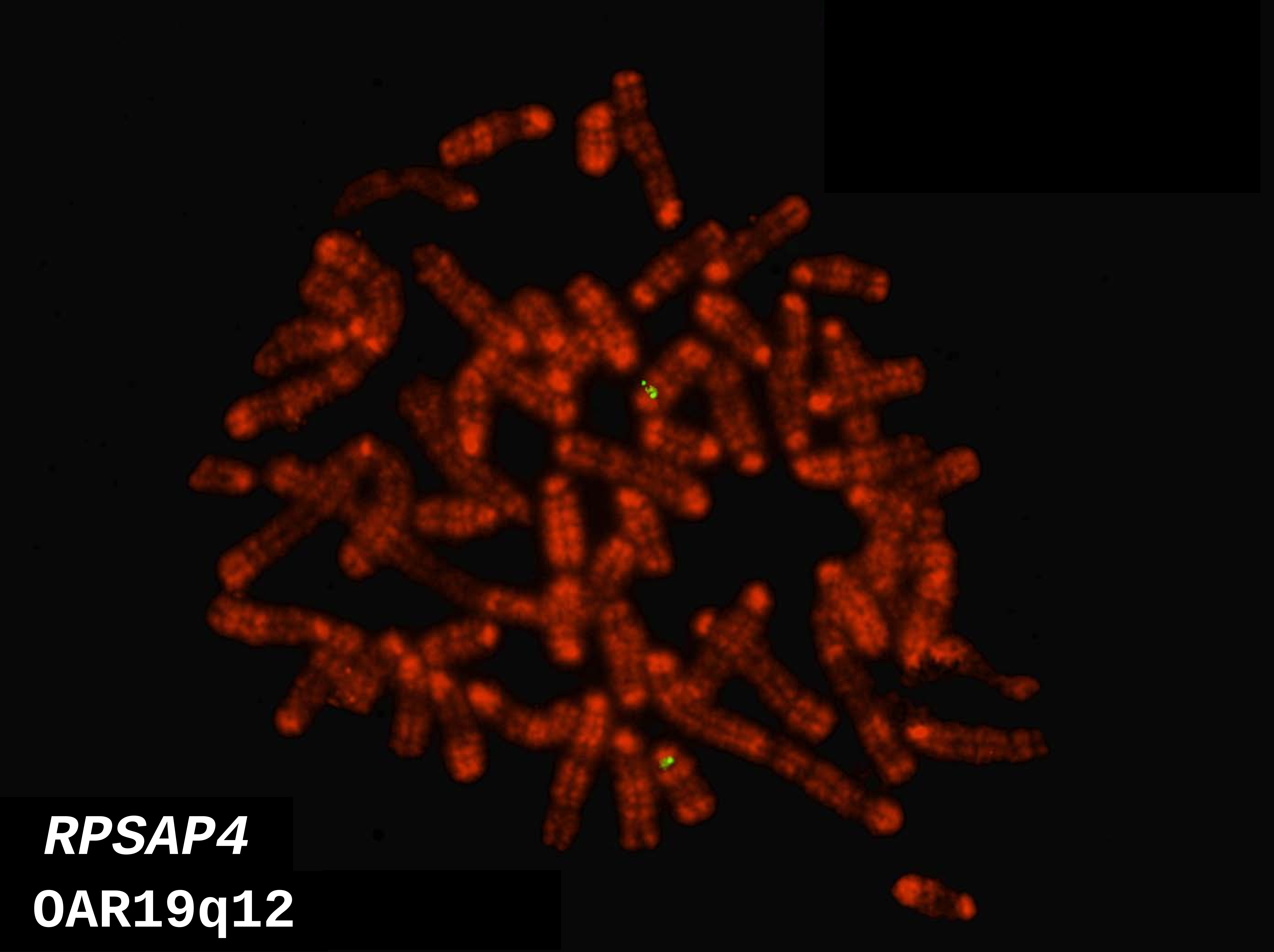
RPSAP4
OAR19q12

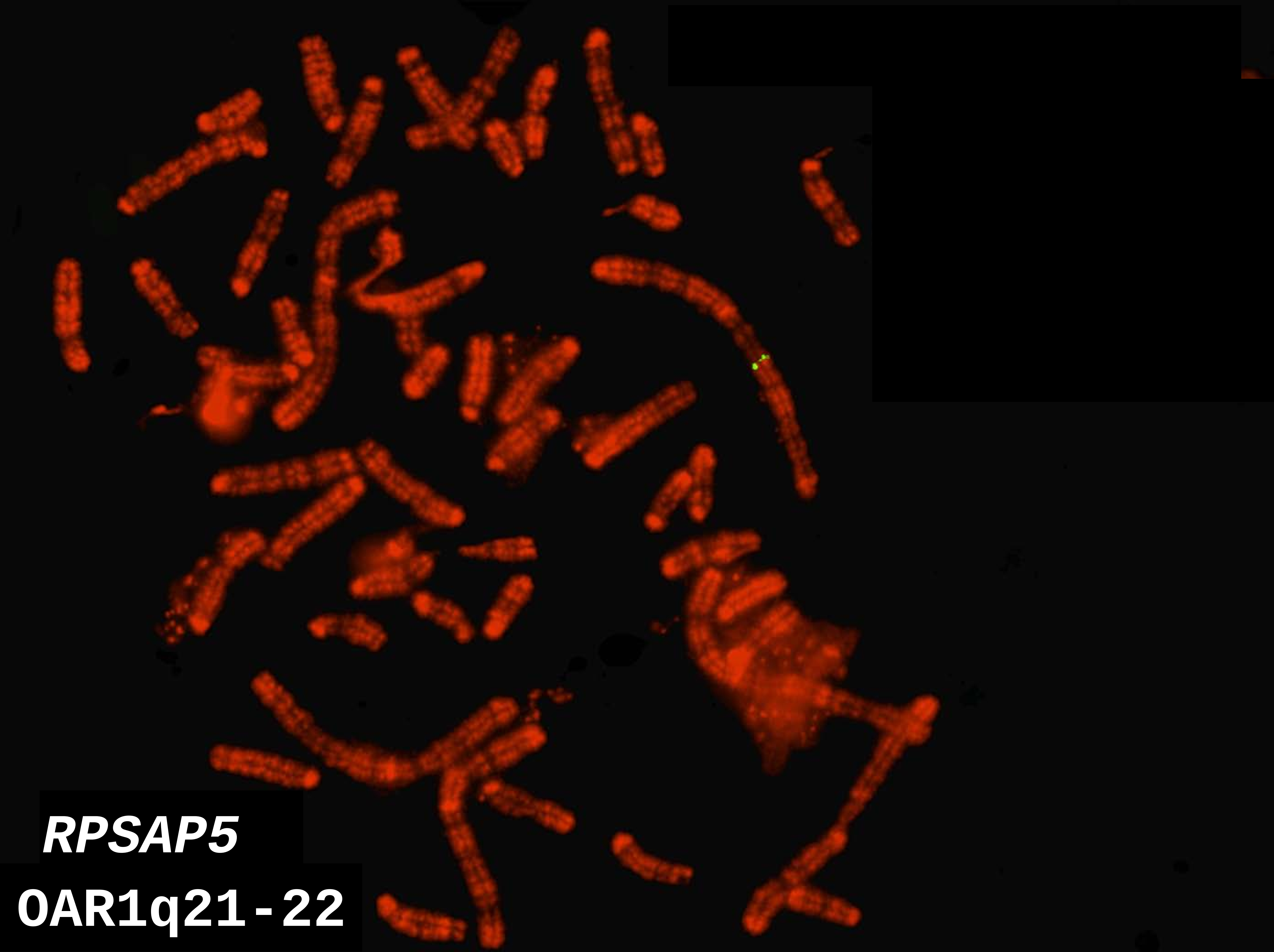
RPSAP5
OAR1q21-22

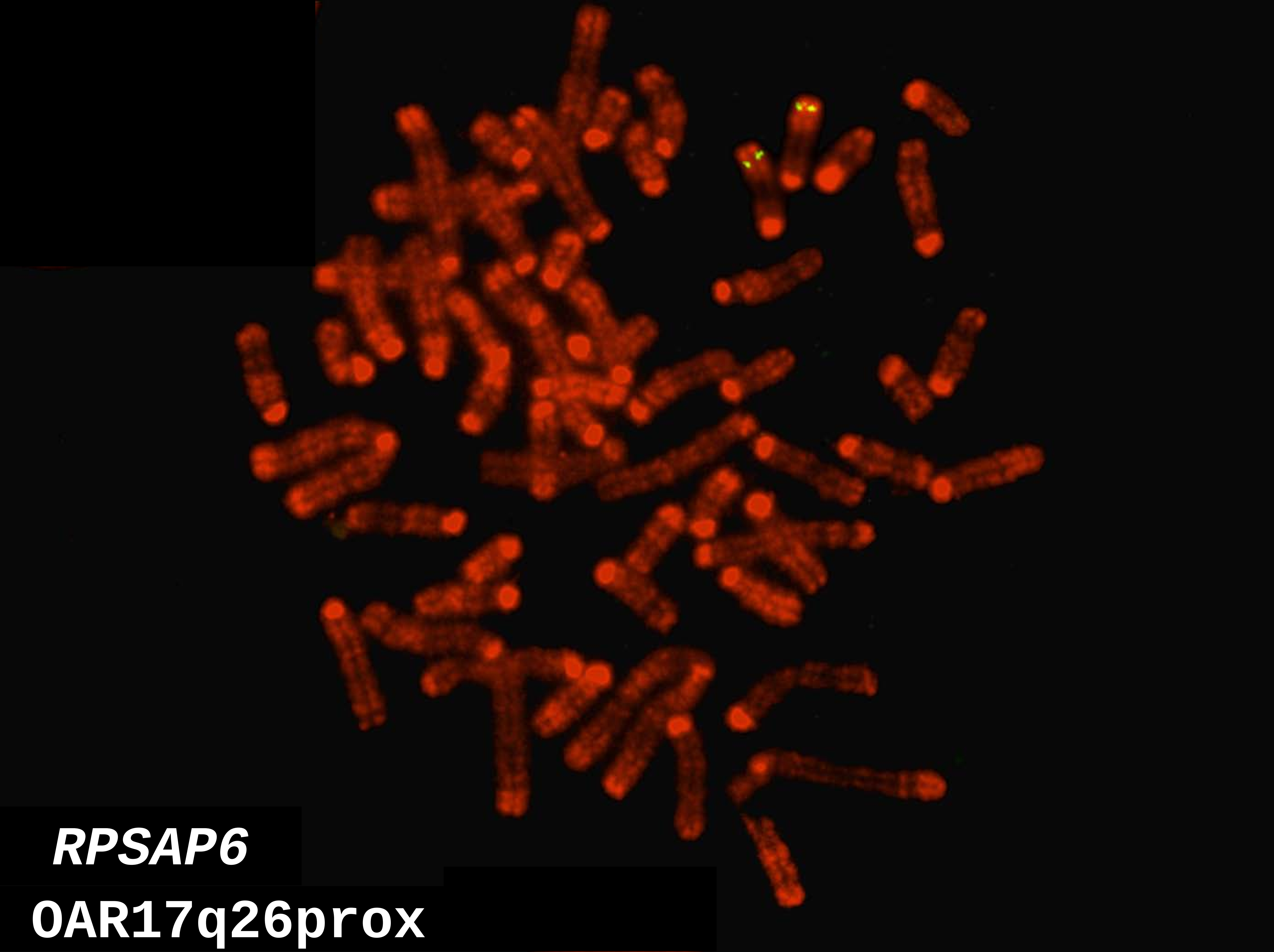
RPSAP6
OAR17q26prox

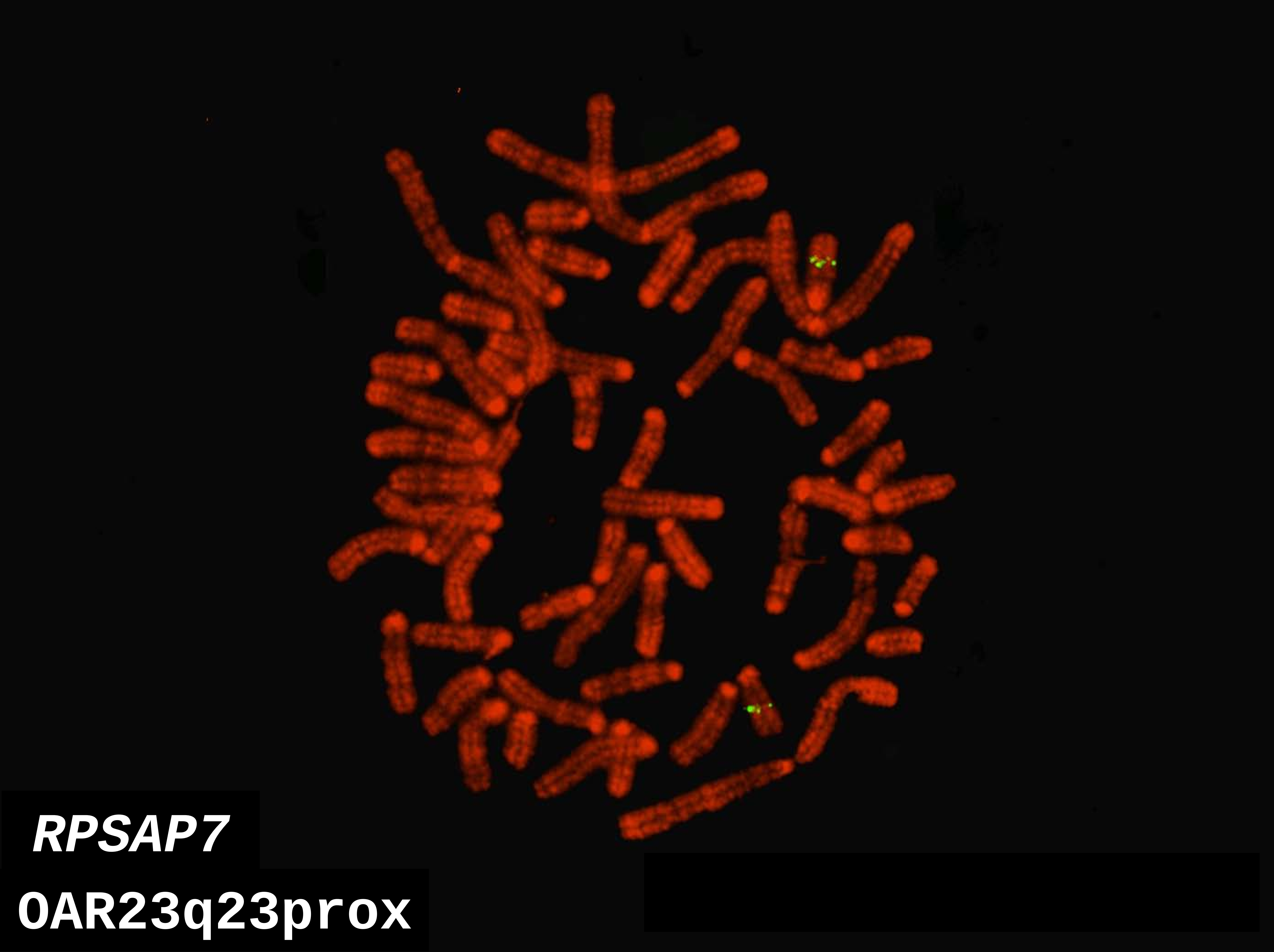
RPSAP7
OAR23q23prox

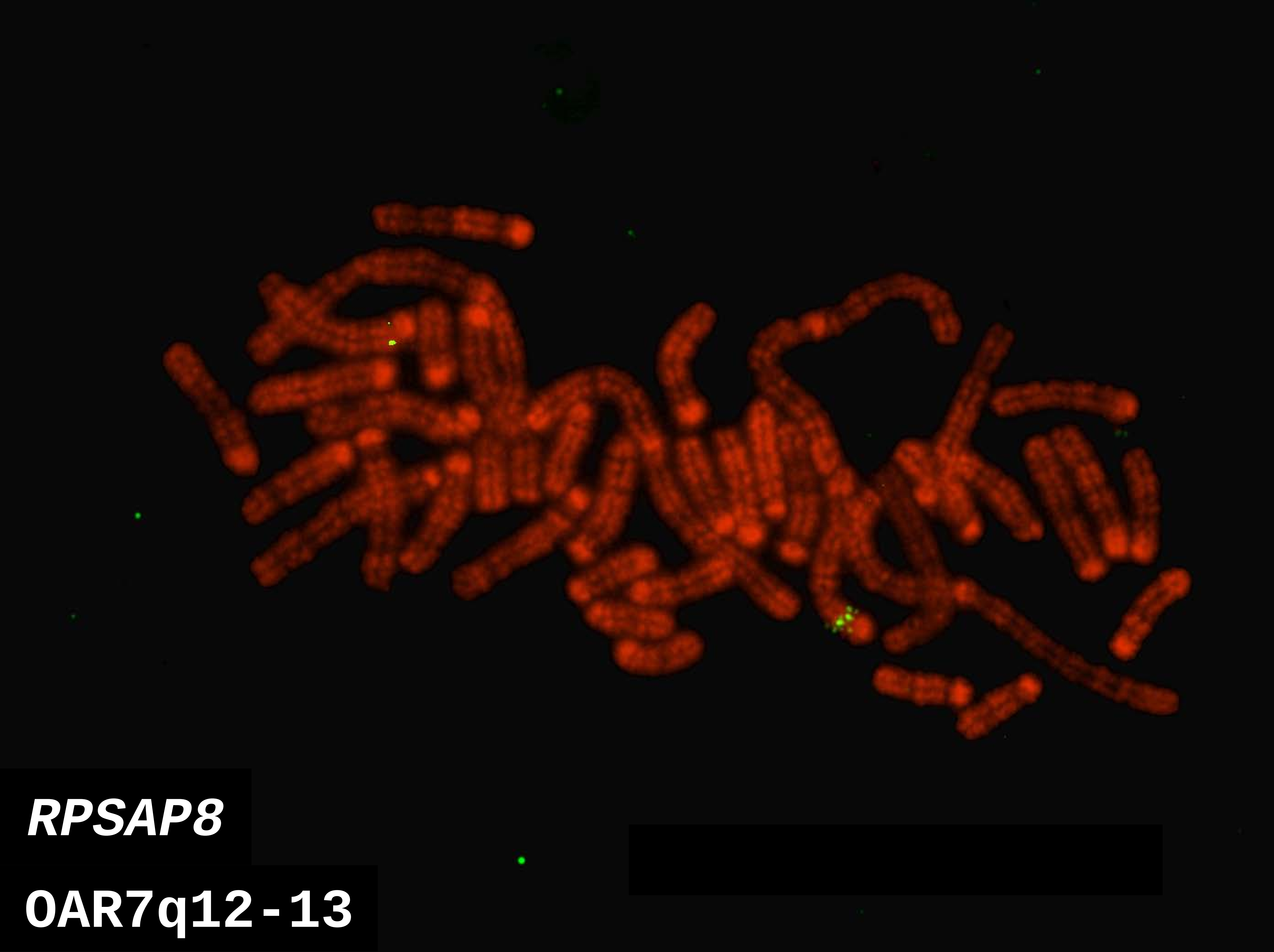
RPSAP8
OAR7q12-13

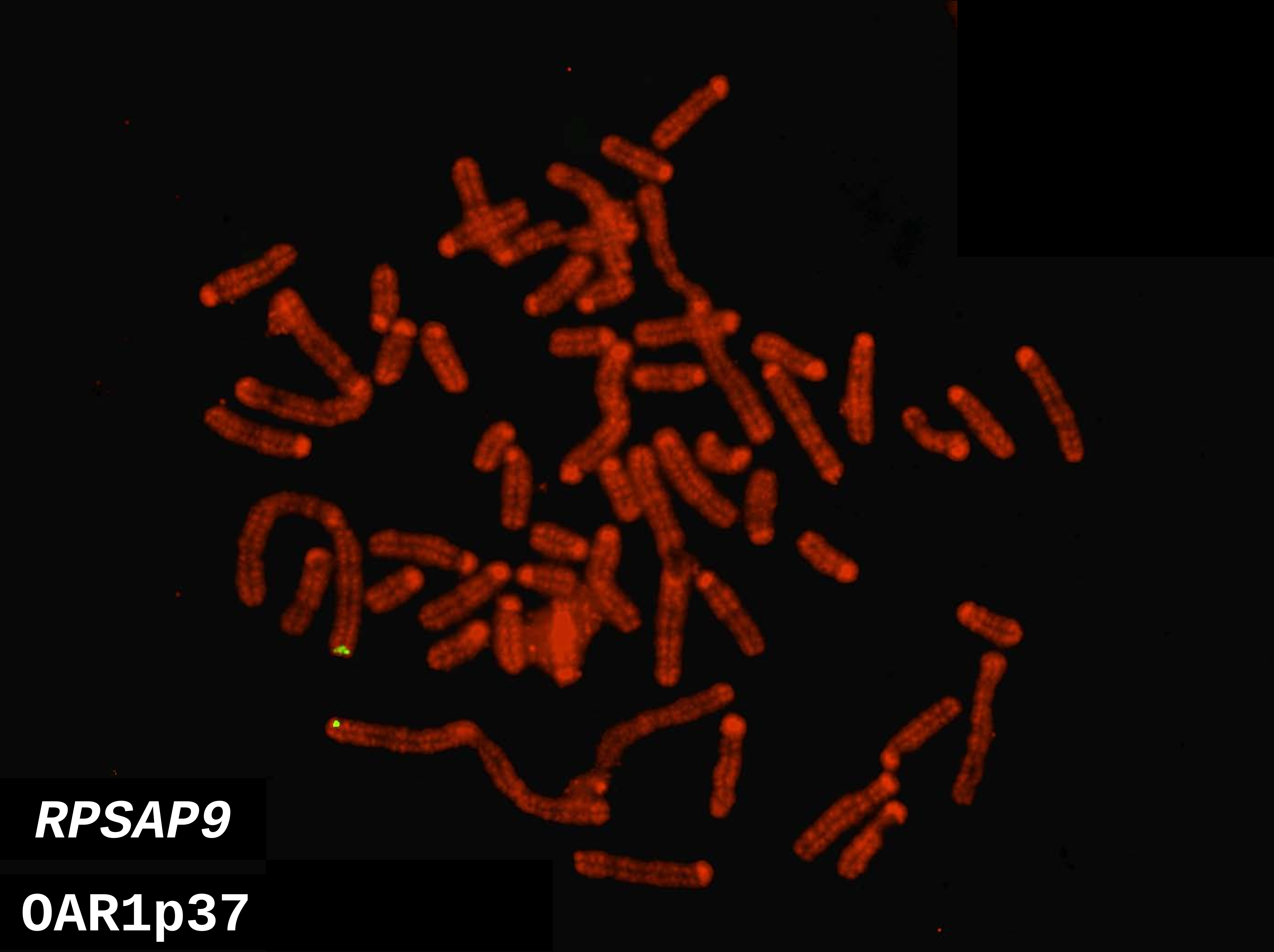

RPSAP9
OAR1p37

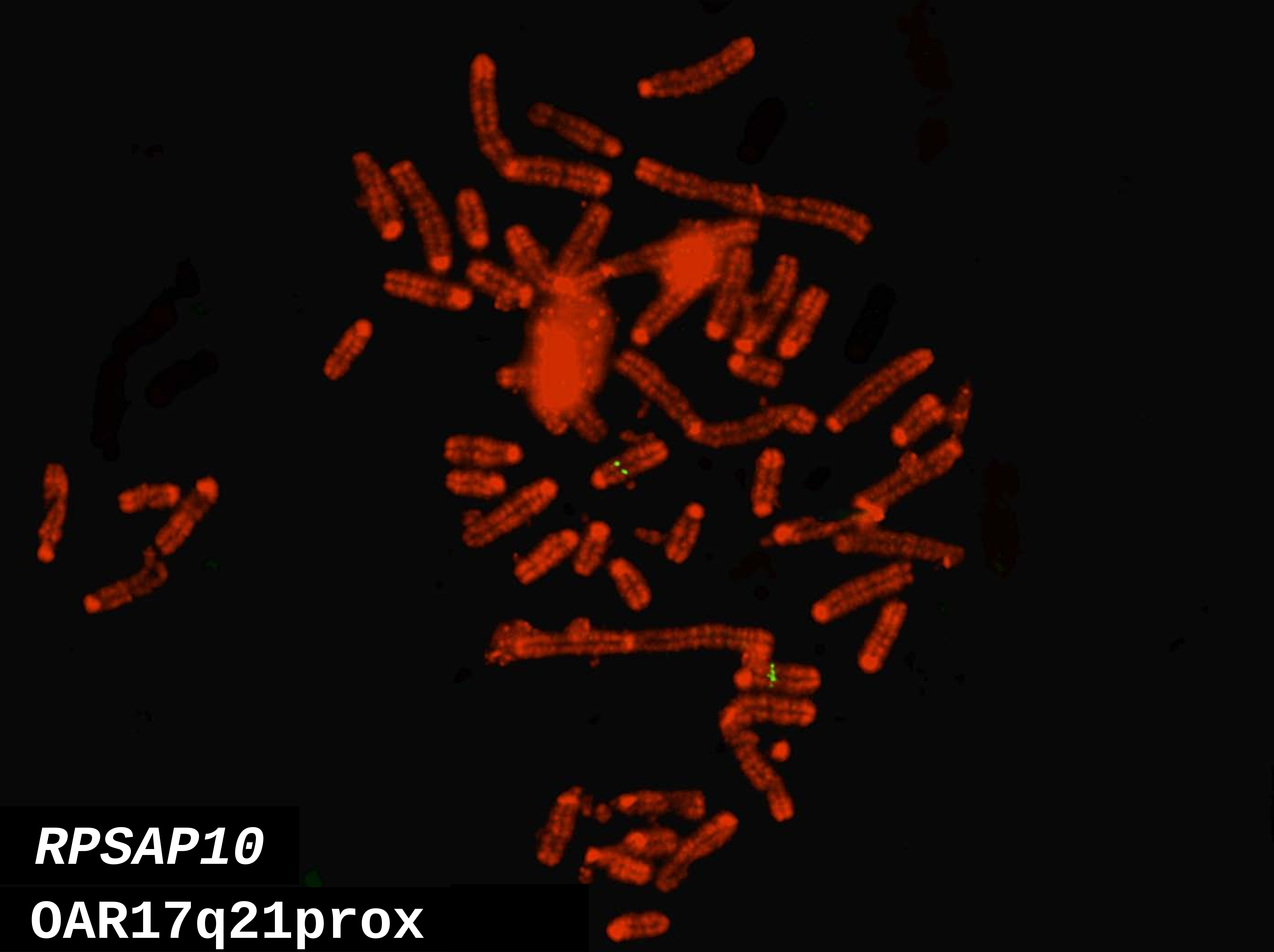
RPSAP10
OAR17q21prox

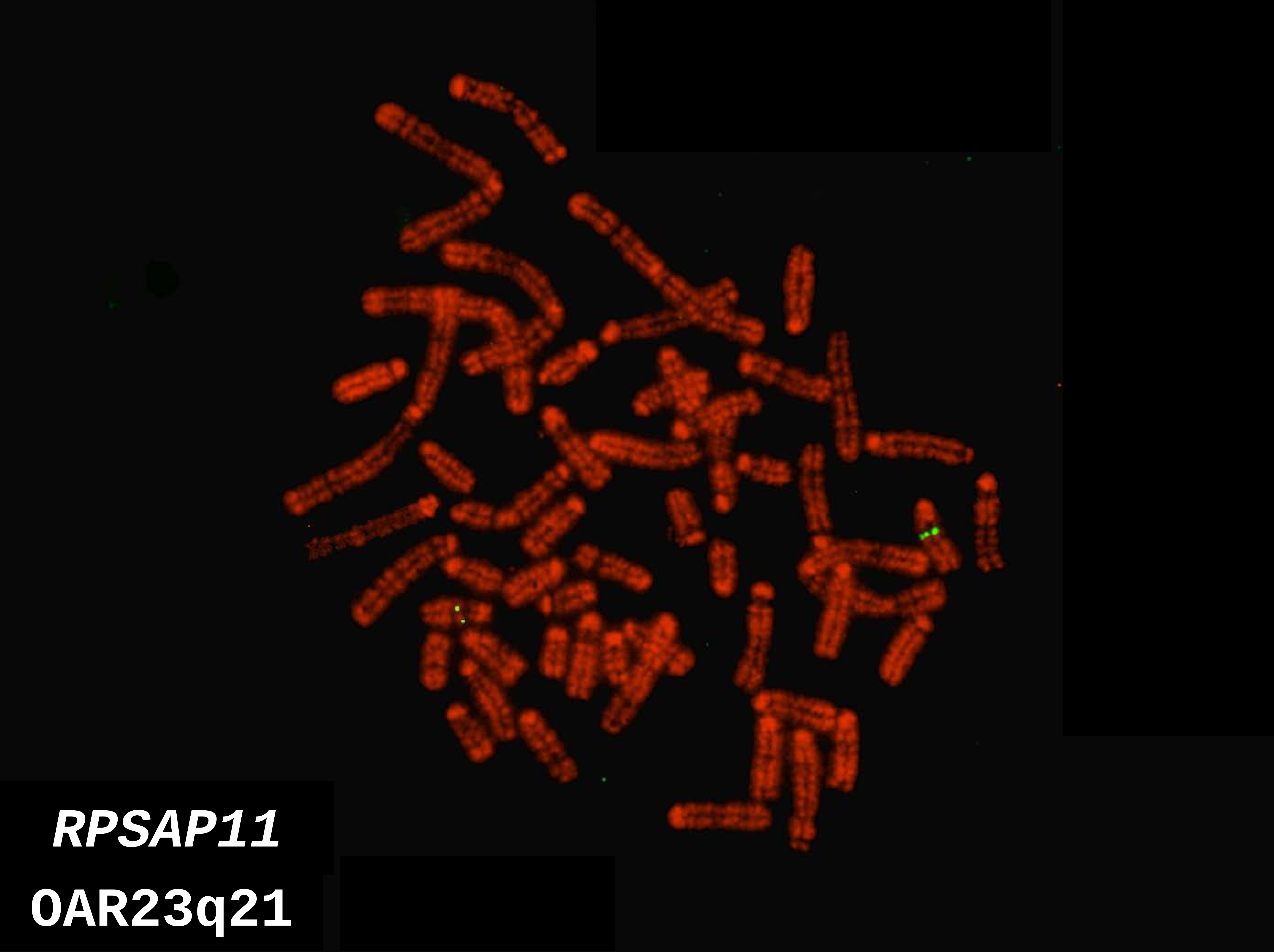
RPSAP11
OAR23q21
