# Supplementary figures and images for: Characterization of the ovine ribosomal protein SA gene and its pseudogenes
Source: BMC Genomics. 2010 Mar 16;11:179. doi: 10.1186/1471-2164-11-179 (PMC2850357; doi:10.1186/1471-2164-11-179)

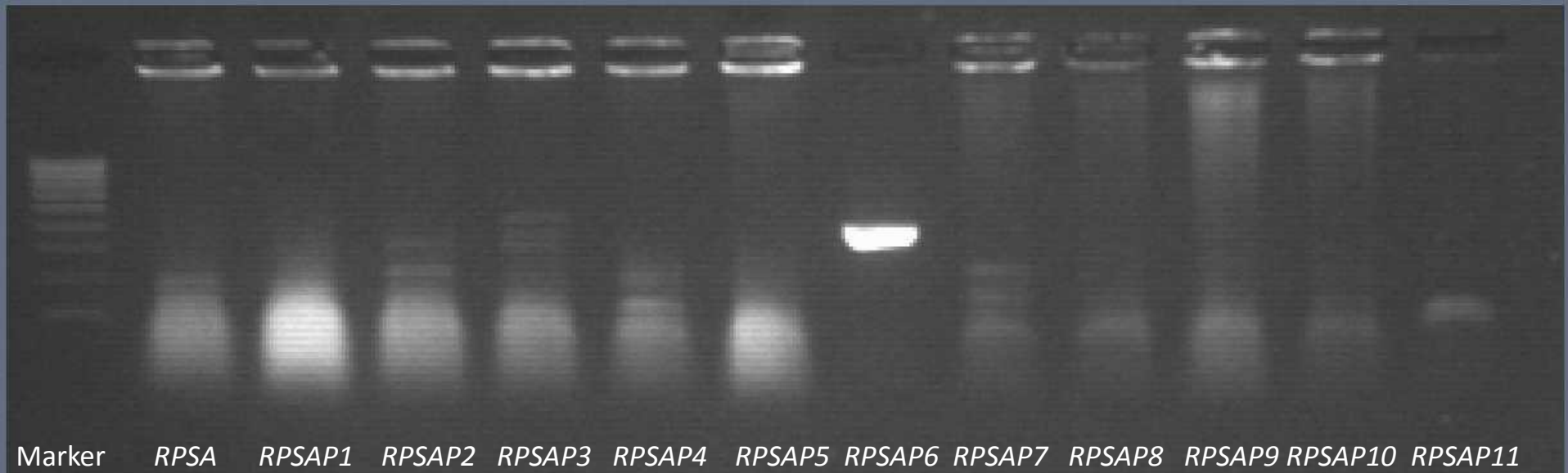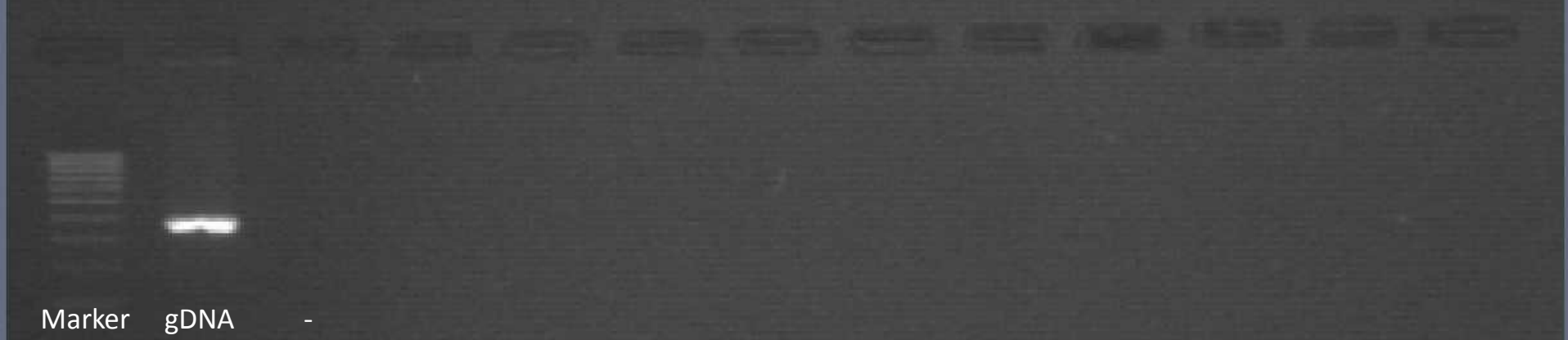

Supplement: Additional file 9 — minus-RT PCR control on RNA isolated from blood. RT-PCR with ACTB primers. Marker (M) is the Hyperladder V (Bioline). Samples are RNA isolated from blood, genomic DNA and water (-). [file 1471-2164-11-179-S9.PDF]

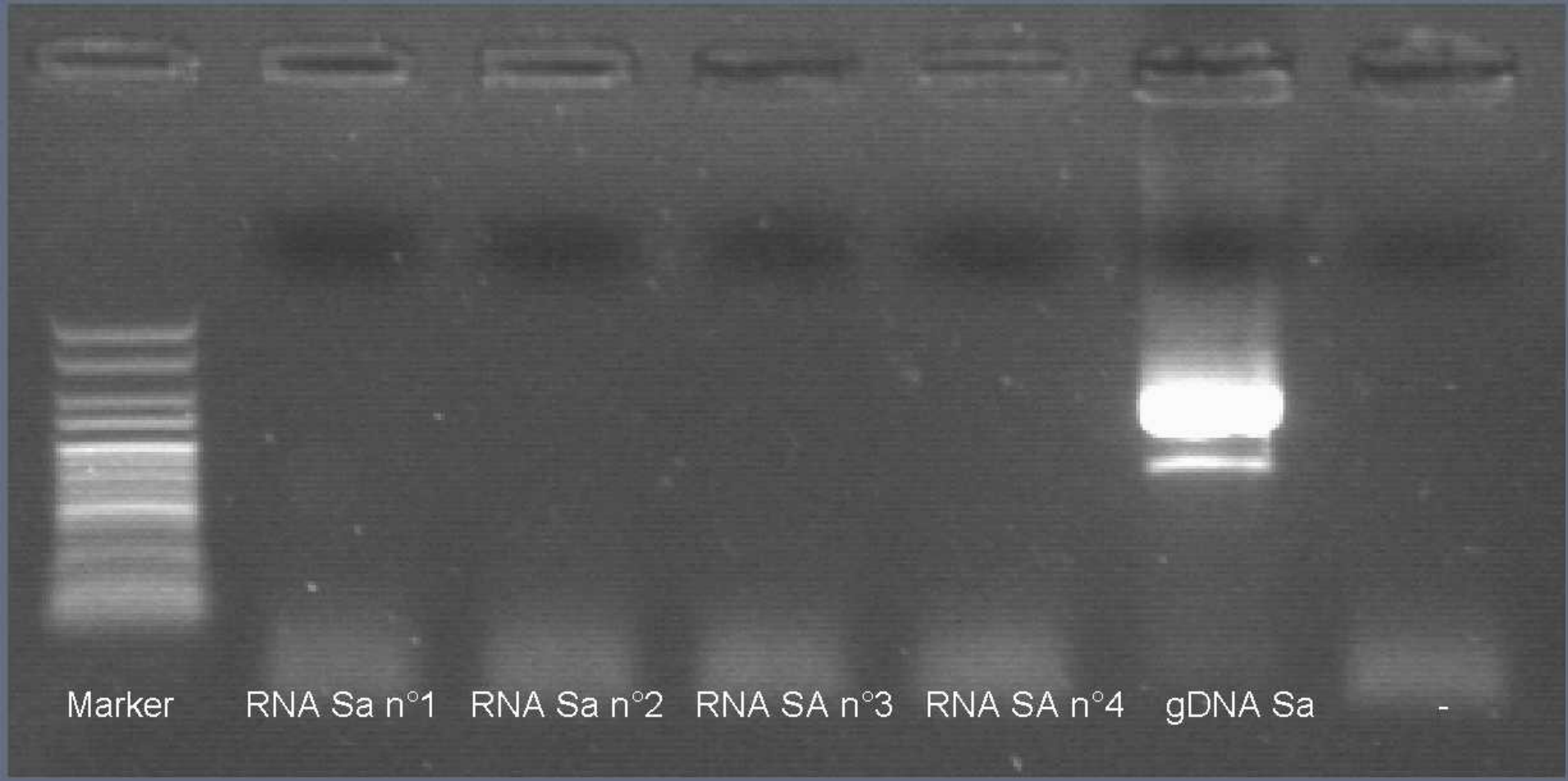

Supplement: Additional file 10 — Specificity of the expression primer for RPSAP6. PCR with expression primer for RPSAP6. Marker (M) is the Hyperladder IV (Bioline). Samples are the respective unique BAC clones of all RPSA family members, genomic DNA and water (-). [file 1471-2164-11-179-S10.PDF]
